# Identification of U2AF(35)-dependent exons by RNA-Seq reveals a link between 3′ splice-site organization and activity of U2AF-related proteins

**DOI:** 10.1093/nar/gkv194

**Published:** 2015-03-16

**Authors:** Jana Kralovicova, Marcin Knut, Nicholas C. P. Cross, Igor Vorechovsky

**Affiliations:** 1 University of Southampton, Faculty of Medicine, Southampton SO16 6YD, UK; 2 Wessex Regional Genetics Laboratory, Salisbury District Hospital, Salisbury SP2 8BJ, UK

## Abstract

The auxiliary factor of U2 small nuclear RNA (U2AF) is a heterodimer consisting of 65- and 35-kD proteins that bind the polypyrimidine tract (PPT) and AG dinucleotides at the 3′ splice site (3′ss). The gene encoding U2AF35 (*U2AF1*) is alternatively spliced, giving rise to two isoforms U2AF35a and U2AF35b. Here, we knocked down U2AF35 and each isoform and characterized transcriptomes of HEK293 cells with varying U2AF35/U2AF65 and U2AF35a/b ratios. Depletion of both isoforms preferentially modified alternative RNA processing events without widespread failure to recognize 3′ss or constitutive exons. Over a third of differentially used exons were terminal, resulting largely from the use of known alternative polyadenylation (APA) sites. Intronic APA sites activated in depleted cultures were mostly proximal whereas tandem 3′UTR APA was biased toward distal sites. Exons upregulated in depleted cells were preceded by longer AG exclusion zones and PPTs than downregulated or control exons and were largely activated by PUF60 and repressed by CAPERα. The U2AF(35) repression and activation was associated with a significant interchange in the average probabilities to form single-stranded RNA in the optimal PPT and branch site locations and sequences further upstream. Although most differentially used exons were responsive to both U2AF subunits and their inclusion correlated with U2AF levels, a small number of transcripts exhibited distinct responses to U2AF35a and U2AF35b, supporting the existence of isoform-specific interactions. These results provide new insights into function of U2AF and U2AF35 in alternative RNA processing.

## INTRODUCTION

Eukaryotic genes contain intervening sequences or introns that are removed from mRNA precursors by a large and highly dynamic RNA–protein complex, termed the spliceosome (1). The spliceosome consists of small nuclear ribonucleoproteins (snRNPs), including the U1, U2, U4/U5/U6 of the major U2 spliceosome and the U11, U12, U4atac / U6atac/U5 of the less abundant U12 spliceosome, and a large number of non-snRNP proteins (1). Spliceosomes assemble on each intron in an ordered manner, starting with recognition of the 5′ splice site (5′ss) by U1 snRNP or the 3′ss by the U2 pathway (2), which involves binding of the U2 auxiliary factor (U2AF) to the 3′ss region to facilitate U2 snRNP recruitment to the branch point (BP) (3,4). U2AF is a stable heterodimer composed of a *U2AF2*-encoded 65-kD subunit (U2AF65), which contacts the polypyrimidine tract (PPT), and a *U2AF1*-encoded 35-kD subunit (U2AF35). U2AF35 interacts with almost invariant AG dinucleotides at 3′ss and stabilizes U2AF65 binding to RNA (5–8).

The *U2AF1* gene is alternatively spliced, giving rise to conserved mRNA isoforms termed *U2AF1a, U2AF1b* and *U2AF1c* (9). In mice, *U2AF1a* is more abundant than *U2AF1b* and contains a highly conserved 67-bp exon 3 in the mRNA whereas *U2AF1b* incorporates exon Ab of the same size (9). The *U2AF1c* isoform includes both exons that introduce a premature termination codon (PTC) in the mRNA, which is targeted by RNA surveillance (9). Both U2AF35a and U2AF35b contain a central U2AF65 recognition domain of the UHM (U2AF homology motif) type flanked by two C3H-type zinc finger (ZF) domains and a C-terminal arginine/serine-rich (RS) region (10–13). Both U2AF35a and U2AF35b interact with U2AF65 and could stimulate its binding to a PPT (9). During evolution, the two U2AF35 proteins have been under high selection pressure, suggesting that they may play specific functions in vertebrates and plants (9,14,15), but their putative functional differences in 3′ss selection are not known.

Although U2AF35 is essential for viability of both yeast and higher eukaryotes (16–19), the 3′ss AG was found to be dispensable for the *in vitro* splicing of pre-mRNAs with strong PPTs (20). Binding of U2AF65 and U2AF35 to weak 3′ss was promoted by splicing activators under splicing conditions (21,22), however, several splicing events assumed to depend critically on U2AF35 did not show any defect under conditions of limited U2AF35 availability *in vivo* (14,23) and some alternative 3′ss responsive to U2AF35 depletion were intrinsically stronger than their nonresponsive counterparts (24). Thus, the distinction between U2AF35-dependent and -independent introns has remained obscure. In addition, overexpression of U2AF65 and depletion of U2AF35 resulted in activation of the same cryptic 3′ss, suggesting that their balance is important for 3′ss selection (24). However, global RNA processing changes in response to U2AF35 depletion or varying ratios of U2AF35/U2AF65 have not been examined.

In this study, we have characterized the transcriptome of human embryonic kidney (HEK) 293 cells lacking U2AF35 and each U2AF35 isoform.

## MATERIALS AND METHODS

### Cell cultures, transfections and library preparations

HEK293 cells were grown under standard conditions in DMEM supplemented with 10% (v/v) bovine calf serum (Life Technologies). For depletion experiments (Supplementary Figure S1), the cells were treated with small interfering RNAs (siRNAs) or splice-switching oligonucleotides (SSOs) targeting splice sites of mutually exclusive *U2AF1* exons 24 h after seeding. Transfections were carried out in 6- or 12-well plates using jetPRIME (Polyplus) according to manufacturer's recommendations. The cells were harvested after 24 and 48 h or received the second hit after 48 h when splitting the cells into new plates. The remaining cultures were harvested 24 and 48 h after the second hit. For RNA sequencing (RNA-Seq), total RNA was extracted using RNeasy Plus (Qiagen) from cells harvested 72 h after the first hit. The NEBNext poly(A) mRNA magnetic isolation module (E7490L) and the Human/mouse/rat Ribo-Zero™ rRNA Removal Kit (Cambio/Epicentre) for RNA were employed according to manufacturers’ recommendations. The libraries were prepared using the NEBNext® Ultra DNA Library Prep Kit for Illumina® (E7370L), size-selected and multiplexed before paired-end sequencing on the HiSeq 2500 Ultra-High-Throughput Sequencing System (Illumina).

siRNAs to downregulate the remaining proteins were as previously described (24 and references therein); the siRNA duplex to heterogeneous nuclear ribonucleoprotein C (hnRNP C) was reported earlier (25).

### Detection of spliced products

Total RNA was transcribed using the Moloney murine leukemia virus reverse transcriptase (Promega) and oligo(dT) primers according to the manufacturer's recommendations. Alternatively spliced U2AF35 exons were visualized by complete *Hinf*I digests of amplified polymerase chain reaction (PCR) products, as described previously (9). Signal intensities of amplified fragments were measured as described (24).

### Immunoblotting

Western blot analyses were carried out as described (24) using antibodies against U2AF35 (10334–1-AP, Protein Tech Group), U2AF65 (U4758, Sigma), actin (ab37063, Abcam), tubulin (ab56676, Abcam) and CAPERα (PA5–31103, Thermo Fisher Scientific). AntiXpress antibodies were purchased from LifeTechnologies (R910–25). Antibodies against PUF60, hnRNP C and hnRNP I (PTB) were a generous gift of Adrian Krainer, Gideon Dreyfuss and Christopher Smith, respectively.

### RNA-Seq analysis

Apart from the knockdown of U2AF35 and its isoforms (Supplementary Table S1), we analyzed RNA-Seq data of previously published knockdown experiments with 11 proteins: heterogenous nuclear ribonucleoprotein (hnRNP) C (Illumina HiSeq 2000) (25), hnRNP A1, hnRNP A2B1, hnRNP H1, hnRNP F, hnRNP M, hnRNP U (Illumina GAII) (26), HOXA1 (Illumina HiSeq 2000) (27), AFF2, AFF3 and AFF4 (Illumina GAIIx) (28). The raw FASTQ data were aligned against the human genome and transcriptome reference using TopHat (v. 2.0.9) (29) and Bowtie (v. 2.1.0) (30) using default stringencies and parameters, except for modification of the UCSC reference (hg19) (31) by introducing the *U2AF1* isoform *s*, which lacks exons Ab and 3. Sequences recognized as originating from mtRNA, rRNA and tRNA were subsequently removed. Analysis of differential exon usage was performed using DEXSeq (1.12.1) (32) and MISO (mixture-of-isoforms) (26). DEXSeq-detected exons were selected based on statistical significance of differential usage (q < 0.05). Unlike DEXSeq, MISO (v. 0.4.9; hg19) (26) computes *p*ercentage of *s*pliced *i*n (psi, Ψ) splice junction-spanning reads and examines their significance using Bayesian factors. The filtering cut-offs were set to default parameters, on the basis of Ψ difference and event significance (ΔΨ > 0.2 and K > 10). Statistically significant events were individually verified in genome browsers to exclude false positives as a result of misannotated transcripts, low expression, overlapping transcripts and apparent misclassifications.

Differential gene and isoform expression between sample sets was analyzed with Cufflinks (v. 2.1.1) (33), which normalizes the reads using a fragments per kilobase of exon model per million reads (FPKM) measure. Gene and isoform expression assessment was aided by the transcriptome reference (hg19, UCSC) with no novel transcript discovery and was followed by CummeRbund (v. 2.2.0) (33) analysis of differential gene and isoform expression (in R environment; v. 3.0.2). Selection of significantly differentially expressed genes was made on the basis of FDR-adjusted *P*-values (q < 0.05). RNA-Seq data for U2AF35 depletion experiments are available at ArrayExpress under the accession number E-MTAB-2682. Finally, gene- and exon-level functional enrichment analyses of differentially expressed events were performed using DAVID (34,35).

### Validation of U2AF35-dependent events

RNA was extracted using TRI reagent, treated with DNase I (LifeTechnologies) and reverse-transcribed as described above. Target transcripts were chosen based on P- and FPKM-values that are shown in full in Supplementary Tables S2 and S3, respectively. PCR primers (Supplementary Table S4) were designed to amplify two or more isoforms with different sizes. Exogeneous transcripts were amplified using RT-PCR with vector primers PL3 and PL4 (36) or their combinations with transcript-specific primers.

### Plasmid constructs

Splicing reporter minigenes were cloned into pCR3.1 (Invitrogen) using primers shown in Supplementary Table S4. IgM minigenes were a generous gift of Martha Peterson, University of Kentucky. Plasmid DNA was extracted using Wizard® Plus SV Minipreps (Promega). Expression constructs of U2AF35 isoforms were described previously (24). PUF60 was subcloned into pCI-neo (Promega) with the Xpress tag at the N terminus, employing the pET28a-PUF60-His construct (24) as a template. All constructs were sequenced prior to transfections to exclude undesired mutations.

### Sequence features of U2AF(35)-dependent exons and introns

Sequences individually validated in genome browsers were examined using algorithms that predict both traditional and auxiliary splice-site recognition motifs. Intrinsic splice-site strength was computed using both maximum entropy and frequency matrix scores (37–40). Prediction of BPs, PPTs and AG exclusion zones (AGEZs) was carried out using a support vector machine (SVM) algorithm (41). *De novo* motif discovery, motif enrichment and motif location analyses were performed using the MEME suite of programmes (42) with sequences flanking differentially used 3′ss, 5′ss and internal exons as the input.

### Measurements of single-strandedness across 3′ss

We computed the PU (probability of unpaired) values for all substrings of high-confidence upregulated and downregulated sequences in their natural context, extending input sequences by 30 nt in each direction. The PU values employ the equilibrium partition function of RNAfold (43) and were as defined previously (44). Input sequences were fixed relative to the position of upregulated and downregulated 3′ss. Their PU values were averaged for each intron position. The means were compared by the Wilcoxon–Mann–Whitney test. Delta PU values are defined here as the difference between mean PU values of upregulated and downregulated exons at the indicated positions.

## RESULTS

### Identification of U2AF(35)-dependent exons

**Figure 1.**
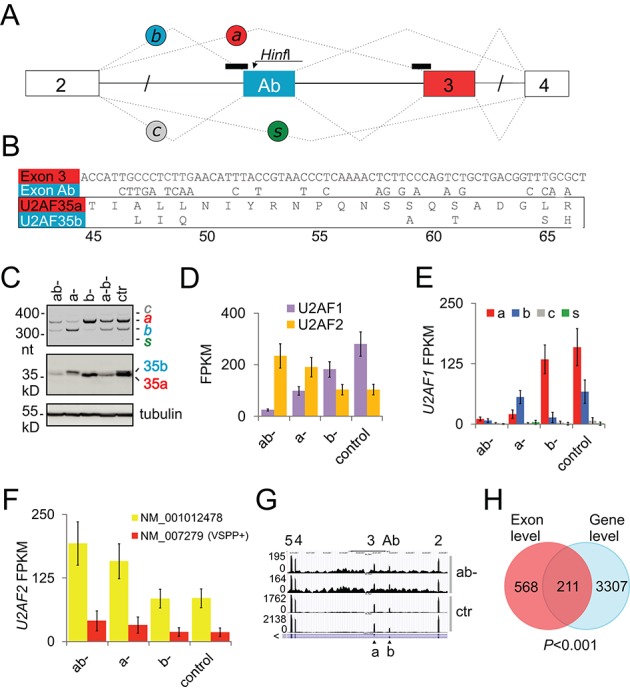
Genome-wide identification of U2AF(35)-dependent exons. (**A**) Alternative splicing of *U2AF1* and location of SSOs. Exons are shown as boxes, introns as horizontal lines, SSOs as black rectangles across 3′ss and spliced products (*a, b, c, s*) as dotted lines. (**B**) Nucleotide (upper panel) and amino acid (lower panel) sequences of alternatively spliced *U2AF1* exons. Amino acids are numbered at the bottom. (**C**) *Hinf*I digested RT-PCR products showing the relative abundance of *U2AF1* isoforms in depleted samples (upper panel) and immunoblot with antibodies against U2AF35 and tubulin (lower panels). ab-, depletion of both isoforms using siRNA U2AF35ab (30 nM); a-, depletion of U2AF35a (60 nM); b-, depletion of U2AF35b (60 nM); a-b-, depletion of U2AF35 using equimolar mixtures of isoform-specific siRNAs; siRNAs were as described (14,23). Ctr, a scrambled control. (**D**) Normalized expression of *U2AF1* and *U2AF2* genes in depleted cultures and controls. FPKM, fragments per kilobase of exon model per million reads. Error bars in panels D–F are 95% confidence intervals. *U2AF1/U2AF2* ratios in ab-, a-, b- and control cultures were 0.09, 0.43, 1.47 and 2.25, respectively. (**E**) Normalized expression of *U2AF1* isoforms. (**F**) Normalized expression of *U2AF2* isoforms. Extra amino acids included in U2AF65 as a result of alternative GC 5′ss usage are in parentheses. (**G**) A genome browser view of exon Ab- and 3-containing isoforms in depleted cells (ab-) and controls (ctr). Exons are numbered at the top and corresponding isoforms are shown at the bottom. Browser views are in the native gene orientation throughout; the 5′>3′ transcriptional orientation is denoted by the > sign. (**H**) Significant sharing of genes identified by DEXSeq (exon-level) and Cufflinks (gene-level) as differentially expressed in ab- cultures versus controls.

We knocked down U2AF35 and each alternatively spliced U2AF35 isoform in HEK293 cells (Figure 1A and B) and examined genome-wide exon/transcript usage by RNA-Seq. We achieved ∼90% depletion with siRNAs targeting both isoforms and a reversal in the relative abundance of U2AF35a and U2AF35b using isoform-specific siRNAs, with a maximum at 72 and 96 h post-transfection (Figure 1C and Supplementary Figure S1). In addition to siRNAs, we employed SSOs targeting 3′ splice sites of alternatively spliced *U2AF1* exons, resulting in a less robust and more delayed response in knockdown levels (Supplementary Figure S1). Using the Illumina HiSeq 2500 platform, we obtained a total of 546 390 339 reads mapped to the annotated human transcriptome and genome, averaging to 68M per RNA sample (Supplementary Table S1). The fraction of *U2AF1* mRNAs ranged between ∼0.001% for depleted samples and ∼0.01% for controls, with isoform-specific knockdowns giving intermediate levels. The range of *U2AF1/U2AF2* ratios varied between 0.1 and 2.3 (Figure 1D and E). Surprisingly, the *U2AF1* depletion was associated with a 2-fold increase of each alternatively spliced *U2AF2* isoform (Figure 1F). The *U2AF1a-*specific depletion led to a lower overall *U2AF1* expression than the *U2AF1b* knockdown while expression of isoforms recognized by RNA surveillance remained low (Figure 1E). The relative abundance of *U2AF1b* slightly increased in cells treated with siRNAs targeting both isoforms compared to untreated cells (Figure 1E and G).

As U2AF35 contacts the 3′ss AG dinucleotide as a part of complexes assembled *ad hoc* on each intron (5–7), we used DEXSeq and MISO algorithms to identify exons differentially used in depleted cells. DEXSeq is based on generalized linear models and relies on biological controls to identify differential exon usage (32), whereas MISO employs a Bayesian approach and splice junction-spanning reads to detect specific alternative splicing events (26). Altogether, DEXSeq identified a total of 484 upregulated and 575 downregulated exons in siRNA U2AF35ab-depleted cultures (termed ab-), with no bias toward either (*P* > 0.05, binomial test), whereas the number of MISO-detected events was ∼60% higher (Supplementary Table S2 and data not shown). Gene-level expression analyses with Cufflinks (33) revealed 1507 upregulated and 2011 downregulated genes (Supplementary Table S3). Overlap of genes with differentially used exons and Cufflinks was highly significant (Figure 1H), suggesting that these exons may contribute to overall gene expression alterations observed in depleted cultures.

### Characterization of global alternative polyadenylation changes induced by U2AF35 depletion

**Figure 2.**
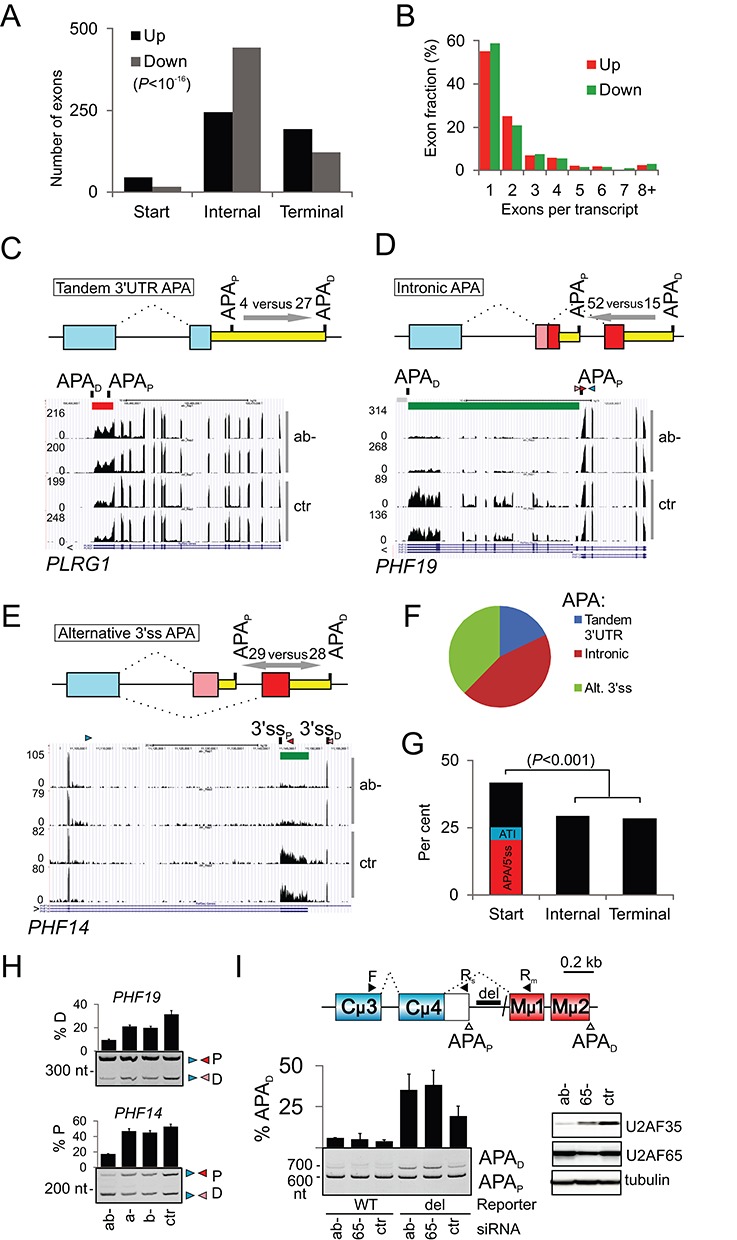
Characterization of global changes in APA induced by U2AF35 depletion. (**A**) Distribution of start, internal and terminal exons upregulated and downregulated in cells depleted of U2AF35. *P*-value was derived from a χ^2^-test for the 3×2 contingency table. *P*-value for the first exons versus the pool of internal and terminal exons was <10^−7^. (**B**) A high proportion of transcripts with ≥2 differentially expressed exons. (**C-E**) APA site usage in the indicated APA categories. Number of proximal and distal APA sites altered in depleted cells is shown above arrows that indicate shifts in APA site usage. APA_P_, APA_D_, proximal and distal polyadenylation sites. Upregulated and downregulated exons are indicated by red and green rectangles throughout. Each category is schematically shown at the top; yellow rectangles are 3′UTRs, blue boxes are constitutive exons, red and pink boxes are alternative exons. Splicing is denoted by dotted lines. (**F**) Frequency distribution of APA categories altered in ab- cells. (**G**) Breakdown of start, internal and terminal exons for Cufflinks-positive genes. APA/5′ss, altered APA or 5′ss of the first intron in ab- cells; ATI, annotated alternative transcription initiation sites altered in ab- cultures. *P*-value was computed as in panel A. (**H**) Validation of intronic/alternative 3′ss APA site usage in two plant homology domain-encoding genes shown in panels D and E. PCR primers are in Supplementary Table S4. (**I**) Control of mouse intronic APA site usage by human U2AF35. Schematics of the mouse IgM minigene with APA sites giving rise to membrane (APA_D_) and soluble (APA_P_) immunoglobulins M (upper panel). Del, deletion of the RNA polymerase II (polII) pausing site (100). The lower panel shows RNA products of the wild-type and deletion-containing IgM minigenes transfected into HEK293 cells individually depleted of each U2AF subunit. Final concentration of U2AF65 siRNA was 40 nM. Immunoblot is to the right.

Among differentially used exons, start and terminal exons were more frequent than expected while internal exons were significantly more common among downregulated than upregulated events (Figure 2A). We observed a similar bias of start/end exons for published RNA-Seq data of cell cultures depleted of other RNA-binding proteins, including hnRNP C, a U2AF65 competitor (25), but not for cells depleted of a DNA-binding factor (Supplementary Table S5). Almost a half of differentially used exons were represented more than once per transcript, consistent with the presence of multiexon segments upregulated or downregulated in depleted cells (Figure 2B). Browser verification of individual events revealed that activation or repression of terminal exons was largely due to the altered usage of previously annotated alternative polyadenylation (APA) sites (45), with intronic APA sites as the most common APA category (Figure 2C–F and Supplementary Table S6). Categorization of 155 APA sites individually confirmed in a genome browser as affected by U2AF(35) depletion and verified against APA repositories revealed that while intron-proximal and -distal APA sites were about equally represented when APA was associated with alternative 3′ss, intronic APA sites promoted in ab- cultures were largely proximal whereas tandem 3′UTR APA sites were biased toward distal sites (Figure 2C–E). Unexpectedly, breakdown of 211 DEXSeq-positive exons in genes that were either upregulated or downregulated in depleted cultures (i.e. Cufflinks-positive) showed preferential involvement of the first exons while terminal and internal exons were about equally represented (Figure 2G). Their individual browser inspection revealed that the excess was attributable to APA and 5′ss of the first introns while altered usage of annotated alternative transcription initiation sites was rare, further supporting a prominent impact of U2AF35 depletion on APA.

Together, these data indicated that APA was a major contributor to the differential exon usage in depleted cells and revealed APA category-dependent shifts of proximal and distal APA sites conferred by a lack of U2AF(35) and/or an increase of U2AF65. They also suggested that a simple distribution of differentially used start, internal and end exons in RNA-Seq depletion experiments could be indicative of the relative importance of a depleted factor for each RNA processing step.

### A high validation rate of DEXSeq-detected alternative RNA processing events

Extensive experimental validation of 82 high-confidence DEXSeq-detected events from the same and independent depletion experiments with the same cell line confirmed 76 exons (Supplementary Table S4, Figure S2), including alternative 3′ss APA in *PHF14* and intronic APA in *PHF19* (Figure 2H). Apart from endogenous RNAs, altered exon inclusion as a result of U2AF(35) depletion was found also for exogenous transcripts (see below), including murine IgM transcripts without the RNA polymerase II (polII) pause site located between proximal and distal APA sites (Figure 2I). As browser verification of MISO-detected events showed a higher false positivity than for DEXSeq (data not shown), their systematic validation was not carried out. However, a high stringency of default DEXSeq settings and a high sensitivity of MISO may provide a higher accuracy in identifying genuine alternative RNA processing events affected by U2AF35 depletion, complementing each other.

### U2AF35 dependency can be explained largely by a lack of the U2AF heterodimer

Apart from altered exon usage seen only in ab- cultures, most transcripts showed a gradient in RNA processing defects, with a hierarchy ab- > a- > b- > controls, mirroring total levels of U2AF35 or U2AF (cf. Supplementary Figure S2A and Figure 1C–E). Individual depletion of each U2AF subunit in HEK293 cells showed that U2AF65 depletion, which reduces U2AF35 levels (14) (Figure 2I), shifted usage of most exons in the same direction as U2AF35 depletion (Supplementary Figure S2B). In *MAPK8IP3* transcripts, however, depletion of U2AF35 promoted inclusion of an 18-nt exon; in contrast, U2AF65 depletion and depletion of U2AF35a failed to activate this exon and led to skipping of the preceding 12-nt exon instead. To understand this phenomenon, we examined these pre-mRNAs in a dose-dependent transfection experiment shown in Supplementary Figure S3A. Interestingly, U2AF65 depletion increased the relative abundance of U2AF35b, suggesting that skipping of the 12-nt *MAPK8IP3* exon in a- and U2AF65- cells was due to the excess of U2AF35b. As U2AF65 depletion reduced U2AF65 more than U2AF35, potentially limiting the amount of the available heterodimer, we estimated residual levels of U2AF in each sample by measuring signal intensity from immunoblots from the same transfection (Supplementary Figure S3A). U2AF levels correlated significantly with the usage of most exons, particularly with those excluded from pre-mRNAs in depleted cells. In contrast, many exons upregulated in U2AF35 depleted cells were not activated in cells lacking U2AF65, and several very small exons, including a 12-nt *MAPK8IP3* exon, did not correlate with U2AF (Supplementary Figure S3B–D and below). Thus, most but not all differential exon usage induced by U2AF35 depletion could be attributed to a lack of the U2AF heterodimer and sequences of these exons should therefore reveal binding signatures of both U2AF subunits.

### Identification of 3′ splice sites altered by U2AF35 depletion

Combined MISO (26) and DEXSeq (32) analyses identified a total of 231 differentially used alternative 3′ss pairs. Their individual inspection in genome browsers confirmed 138 pairs of 3′ss, with 93 intron-proximal sites promoted and 45 repressed in ab- cultures (Supplementary Table S7). Only 51/138 sites (37%) promoted in ab- cultures were intrinsically stronger than their competing counterparts (binomial test, two-tail *P* = 0.003; Supplementary Table S9). We also observed a significant lack of canonical cytosine at position −3 and guanine at position +1 relative to upregulated proximal 3′ss (Supplementary Figure S4A and B). Both positions contribute substantially to the 3′ss strength (37).

Since over a third of APA sites altered in depleted cells was associated with annotated alternative 3′ss usage (Figure 2E and 2F, Supplementary Table S6), we tested if their intrinsic strength contributes to APA selection. Analysis of browser-verified 57 pairs of alternative APA 3′ss showed that unlike alternative 3′ss not associated with APA, intrinsically weaker sites were not preferred (28 stronger versus 29 weaker in ab- cultures). Likewise, we observed no enrichment of weaker sites when comparing proximal upregulated and downregulated APA 3′ss with APA 3′ss of terminal exons. Although the number of APA-associated alternative 3′ss was smaller than the number of non-APA 3′ss pairs, this result is consistent with an additional function of U2AF in APA control that is independent of interactions with alternative 3′ss of internal exons.

### U2AF(35) depletion can influence 5′ splice-site usage

U2AF35 contacts 3′ss AG (5–7), but we identified at least 32 alternative 5′ss pairs influenced by U2AF35 depletion. Eleven of them were tested using RT-PCR and 10 were confirmed (Supplementary Tables S4 and S8; Figure S5A–G). Over a third of these 5′ss (*n* = 11) were located in the first introns. As for 3′ss, proximal (26 versus 6) and weaker (19 versus 13) 5′ss were activated more often in depleted cultures than their distal and stronger counterparts. Activation of cryptic 3′ss was occasionally accompanied by cryptic 5′ss activation of the same exon (Supplementary Figure S5H). Because intronic APA sites compete with upstream 5′ ss (46), we also determined the intrinsic strength of the first 5′ss upstream of 67 intronic APA sites affected by U2AF35 depletion. Their average scores tended to be lower (*P* = 0.12; Supplementary Table S9) than those of authentic counterparts of aberrant 5′ss, which were used as controls (40).

### U2AF35 depletion activates exons with longer AGEZs and PPTs

**Figure 3.**
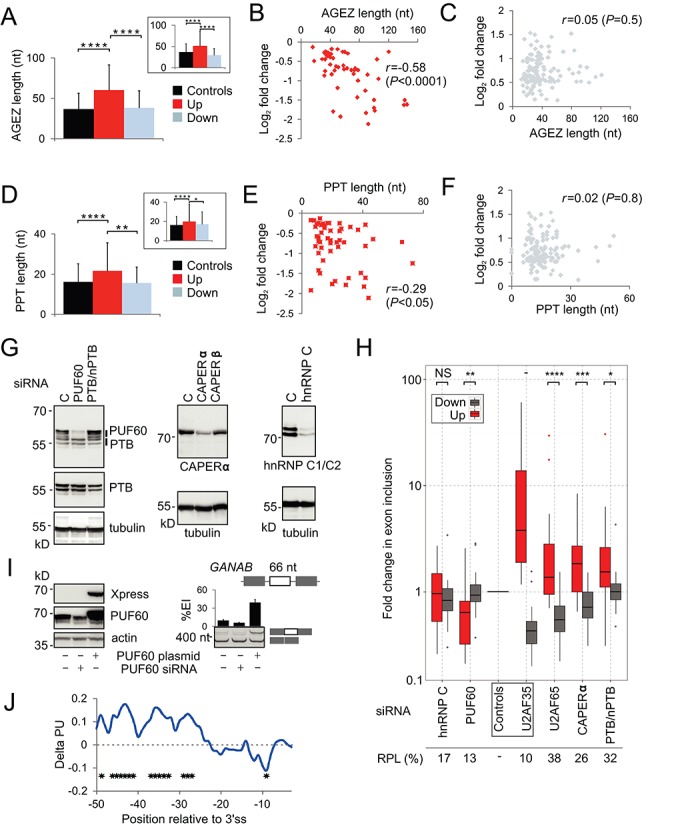
Structural and functional characterization of U2AF(35)-dependent 3′ss. (**A, D**) AGEZ (A) and PPT (D) length of alternative 3′ss (inset) and internal exons affected by U2AF35 depletion. Columns show means, error bars denote SDs. The number of each event is in the legend to Supplementary Figure S8. PPT length was computed for BPs with the highest SVM scores (41). *P*-values were derived from t-tests; ****P < 0.00005; **P < 0.005. AGEZ and PPT length correlated with the expression change of upregulated (**B, E**) but not downregulated (**C, F**) exons; *r*, Pearson correlation coefficient. (**G**) Immunoblots prepared from lysates from HEK293 cells depleted of poly(Y)-binding proteins (indicated at the top). Antibodies are shown at the bottom or to the right. (**H**) Functional antagonism and synergism of Y-binding proteins and U2AF. Exon inclusion levels of each transcript are in Supplementary Figure S11; RT-PCR primers are in Supplementary Table S4. Residual protein levels (RPL) were estimated from immunoblots shown in panel G. The Y-axis is on a log_10_ scale. Average changes between inclusion levels of upregulated and downregulated exons were compared by the Wilcoxon-Mann-Whitney test (*P < 0.05; **P < 0.005; ***P < 0.0005; ****P < 0.00005; NS: not significant; -: not tested). (**I**) Opposite effects of PUF60 depletion and overexpression on a *GANAB* exon. Immunoblots with the indicated antibodies are to the left and RT-PCR to the right. The *GANAB* minigene is schematically shown at the top; alternative exon is denoted by a white rectangle. Exogeneous RNA products were amplified by primers PL3 and PL4. Error bars are SDs of duplicate transfections. (**J**) Positional differences in unpaired probabilities upstream of U2AF(35)-activated and repressed exons. Positive delta PU values signify a higher average single-strandedness of upregulated exons in the optimal BP location and further upstream as compared to downregulated exons whereas negative values reveal their tendency to engage in local base-pairing interactions closer to 3′ss. Stars denote positions with *P*-values <0.05.

Examination of 204 browser-verified internal exons that excluded multiple-exon regions associated with APA sites revealed that U2AF35 depletion promoted both exon inclusion and skipping as well as intron splicing and, sporadically, intronization within large exons (Supplementary Figure S6A and B). Interestingly, exon activation in depleted cells often occurred in weak, incompletely removed introns (Supplementary Figure S6C). U2AF(35)-sensitive exons were largely alternatively spliced and had sequence features typical of alternative exons (Supplementary Figure S7 and Table S10). Importantly, upregulated exons had a significantly longer AGEZ than downregulated or control exons (Figure 3A), but did not show a comparable decrease of other purine dinucleotides upstream of 3′ss (Supplementary Figure S8A). The AGEZ length correlated with the expression change of upregulated but not downregulated exons (Figure 3B and C). Upregulated exons had also longer PPTs for the best predicted BP although the associated *P*-values were lower than for the AGEZ (Figure 3D). The PPT length also correlated with the change in usage of upregulated exons (Figure 3E and F). A specific lack of AGs and longer AGEZs/PPTs was found also upstream of alternative 3′ss upregulated in ab- cultures (insets in Figure 3A and D and Supplementary Figure S8B) and this tendency was observed also for as few as 57 alternative 3′ss leading to APA (Supplementary Figure S9, Figure 2E).

Interestingly, the lack of AGs upstream of upregulated exons was associated with adenine depletion between position −17 and −38, which approximately corresponds to the optimal BP location (20,47), whereas guanine depletion was more widely distributed (Supplementary Figure S10A and B). Instead, upregulated exons showed enrichment for adenine at positions −3 to −9 while uridine tended to show the opposite, with enrichment between −17 and −38 and depletion closer to the 3′ss. Conversely, downregulated exons showed adenine enrichment in the optimal BP region, particularly in smaller exons (Supplementary Figure S10C–E), and uridine enrichment closer to 3′ss. We also searched for additional known and unknown motifs in sequences flanking U2AF(35)-sensitive junctions using the MEME suite of programs (42), however, no significant hits were found.

We conclude that 3′ss upregulated in ab- cells have longer AGEZs/PPTs, adenine depletion in the optimal BP location and enrichment closer to 3′ss, with uridines showing the opposite pattern. This arrangement moves PPTs further upstream of upregulated exons as compared to their downregulated counterparts. The observed widespread repression by U2AF(35) could thus reflect a lack of AG dinucleotides upstream of 3′ss, which are generally repressive when located downstream of BPs (20,48,49), and/or longer, more upstream PPTs, which may bind exon-repressive PPT-binding proteins (25,50). Because these sequence characteristics are likely to influence secondary structure formation across 3′ss, this group of exons should provide a powerful tool to study regulation of 3′ss by RNA-binding proteins and RNA folding.

### Exons repressed by U2AF(35) are stimulated by PUF60 and inhibited by CAPERα

To examine the role of U2AF-related proteins in usage of U2AF-dependent exons, we measured inclusion of 44 exons in HEK293 cells depleted of PUF60, CAPERα (RBM39), CAPERβ (RBM23) and two other Y-binding proteins (Figure 3G). PUF60 and U2AF were individually capable of protecting the 3′ss AG in footprinting experiments (51), but the exact function of CAPERα/β in recognition of 3′ss or U2AF-dependent exons is unknown. We also depleted hnRNP A1, which allows U2AF to discriminate between pyrimidine-rich RNA sequences followed or not by a 3′ splice-site AG (52), and DEK, which facilitates the U2AF35-AG interaction and prevents binding of U2AF65 to pyrimidine tracts not followed by AG (53) (data not shown). Remarkably, the majority of exons upregulated in cells depleted of U2AF35 were downregulated in cells depleted of PUF60 (Figure 3H and Supplementary Figure S11). In contrast, CAPERα, and to a lesser degree PTB, showed synergism with U2AF(35) for this group of exons (Figure 3H). A significant directionality of hnRNP C, DEK and hnRNP A1 could not be established with this sample size (Figure 3H and data not shown). To confirm that PUF60 stimulates exons repressed by U2AF, we measured exon inclusion of a minigene reporter transfected into HEK293 cells lacking or overexpressing PUF60 (Figure 3I). We found that cells lacking PUF60 showed increased skipping of this exon, whereas the PUF60 overexpression increased its inclusion in the mRNA. Thus, U2AF(35)-induced exon usage was predictive of responses to other Y-binding proteins, revealing the connection between antagonism and synergism of U2AF-related proteins and characteristic 3′ss organization described above.

### Unpaired regions upstream of U2AF(35)-activated and -repressed exons

Because characteristic changes in nucleotide frequencies upstream of U2AF(35)-dependent exons (Supplementary Figure S10) are likely to affect formation of RNA secondary structure, which can influence 3′ss utilization (54,55), we computed position-specific probabilities of RNA single-strandedness (44) for high-confidence upregulated and downregulated internal exons. Remarkably, upregulated exons showed on average significantly higher unpaired probabilities at most positions between −25 and −50 than downregulated exons while a lower single-strandedness was observed closer to their 3′ss (Figure 3J). This finding suggests that intramolecular base-pairing interactions over relatively long distances upstream of 3′ss control exon repression and activation by U2AF and, most likely, by U2AF-related proteins that showed functional antagonism and synergism with U2AF and bind single-stranded RNA (Figure 3H).

### U2AF(35) preferentially regulates nuclear proteins involved in RNA binding

**Figure 4.**
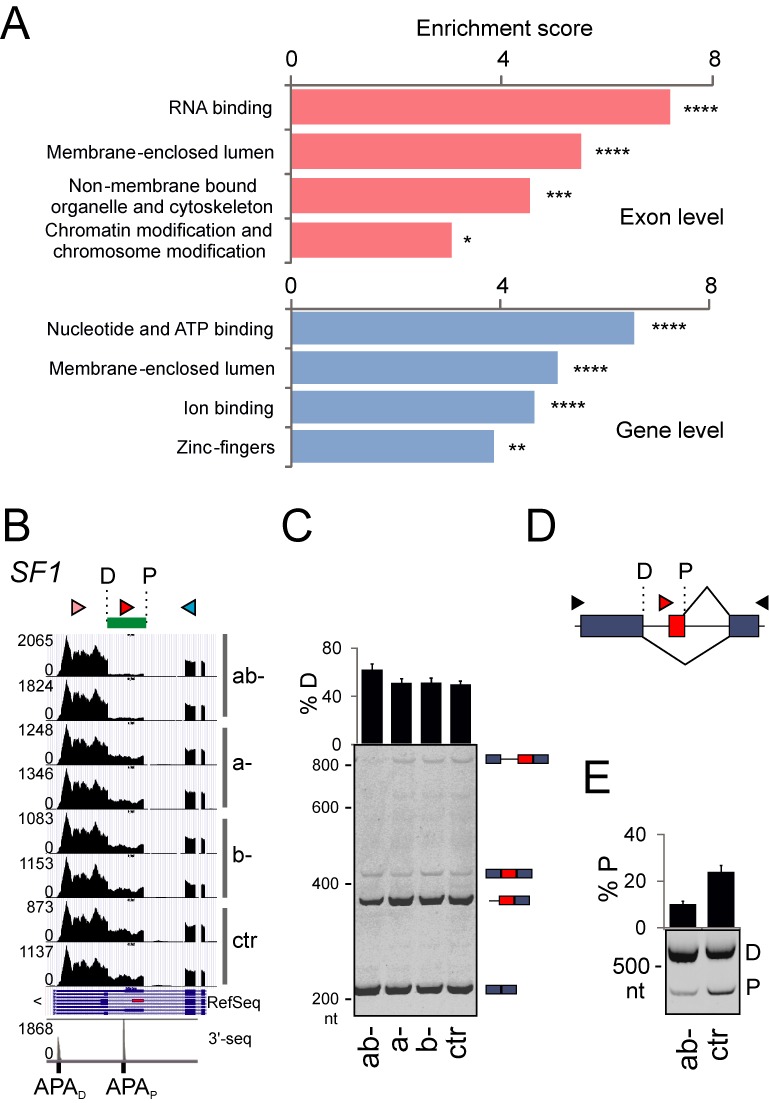
U2AF(35)-regulated exons are overrepresented in genes encoding RNA-binding proteins. (**A**) Functional enrichment analysis using DAVID (35). Asterisks denote the FDR significance (*P < 0.05; **P < 0.005; ***P < 0.0005; ****P < 0.00005). (**B**) Regulation of alternative 3′ss site usage of *SF1* by U2AF(35). P, D, proximal and distal 3′ss of the last *SF1* intron. Arrowheads denote RT-PCR primers (Supplementary Table S4) used in panel C. The last track shows unified 3′-seq coverage from multiple tissues with the location of two APA sites (45). Supplementary Figure S12 shows their usage in various cell types; the proximal APA site is used only weakly in HEK293 cells. (**C**) Activation of distal 3′ss *SF1* in depleted cells. RNA products are schematically shown to the right. Error bars denote SDs. (**D**) Schematics of the *SF1* minigene. Arrowheads show primers used for RT-PCR in panel E. (**E**) RNA products of the *SF1* minigene. Transient transfections were into HEK293 cells (mock)-depleted of U2AF35.

Functional enrichment analysis (35) of exons/genes differentially used in U2AF35 depleted cells showed that they were enriched in proteins involved in RNA/nucleotide binding, respectively (Figure 4A). Figure 4B shows a single example of alternative 3′ss in a known RNA-binding factor *SF1*. These 3′ss are responsible for production of BP-binding SF1 proteins with variable C-termini (56) and are associated with tissue-specific APA sites (Supplementary Figure S12). These proline-rich regions interact with PRPF40A (57), a component of the splicesomal E complex (58). Depletion of U2AF35 was associated with upregulation of *SF1* mRNA and promotion of the distal 3′ss (Figure 4B and C). Transfection of the *SF1* splicing reporter constructs into ab- cells confirmed repression of the proximal site (Figure 4D and E). Thus, U2AF(35) regulates the length of *SF1* 3′UTR and, potentially, its PRPF40A interactions.

**Figure 5.**
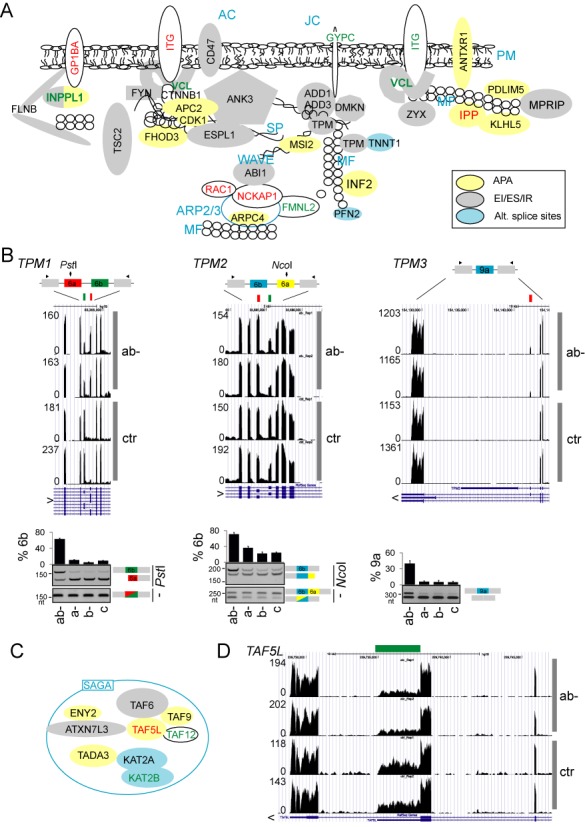
Examples of U2AF(35)-regulated genes involved in cytoskeleton organization and chromatin modification. (**A**) Exon-centric regulation of actin dynamics. Protein products are drawn as colored shapes in a cellular context. Red and green text shows genes that were upregulated and downregulated in ab- cells, respectively; blue text denote protein complexes or subcellular structures: PM, plasma membrane; MF, microfilaments; SP, spectrin; AC, ankyrin complex; JC, junctional complex. EI, exon inclusion; ES, exon skipping; IR, intron retention. Actin monomers are schematically shown as small circles. ITG shapes denote multiple integrins upregulated and downregulated in ab- cultures (Supplementary Table S3). (**B**) Identification of U2AF(35)-sensitive exons in the tropomyosin genes (upper panels) and their validation by RT-PCR (lower panels). Restriction enzymes to establish the identity of mutually exclusive exons are indicated to the right; small fragments of digested products are not shown. Alternative exons are coloured, arrowheads denote PCR primers. (**C**) Components of the SAGA complex influenced by U2AF35 depletion. A legend for colored shapes is in panel A. (**D**) A genome browser view of a differentially used APA in a representative SAGA transcript. Additional SAGA transcripts are shown in Supplementary Figure S5F and S5G.

In addition to RNA binding, differentially expressed exons/genes were enriched in proteins involved in cytoskeleton organization, chromatin modification and proteins found in the organelle lumen (Figure 4A). Figure 5A gives a summary of exons in transcripts involved in actin dynamics. Tropomyosin genes (*TPM*s), which control function of actin filaments in a tissue-specific manner (59), serve as the most prominent examples. In ab- cultures, exon 6a of *TPM1* and *TPM2* was repressed and exon 6b, which has a long PPT in both genes, was activated (Figure 5B). Isoforms containing exon 6b have lower calcium sensitivity than isoforms with exon 6a, which may be required for a specific interaction with troponin (60). *TNNT1*, a gene coding for a slow skeletal muscle troponin T, sustained a cryptic 3′ss activation upon U2AF35 depletion (data not shown). Besides *TPM1/2*, exon 9a of *TPM3* was upregulated in depleted cells as well as the *TPM4* transcripts (Figure 5B and Supplementary Table S3). *TPM3* isoforms expressing exon 9a are more widely distributed in tissues than isoforms containing exon 9c (61), suggesting that U2AF(35) restricts tissue expression of γ-tropomyosin.

Finally, Figure 5C shows U2AF(35)-induced alterations of exon usage in eight genes coding for the histone modifying SAGA (Spt-Ada-Gcn5 Acetyltransferase) complex components, including *KAT2A* and *KAT2B*. The U2AF35 depletion promoted the use of a distal 5′ss in *KAT2A* (Supplementary Figure S5F), modifying the balance of alternatively spliced GCN5 isoforms and, most likely, histone acetyltransferase activity. 5′ss selection was influenced also in a GCN5 paralog *KAT2B* (PCAF; Supplementary Figure S5G). The *TADA3* gene, which encodes the GCN5 interaction partner, sustained activation of the proximal APA site upon depletion. In contrast, a proximal APA was repressed in *TAF5L*, a component of the SAGA architecture module (Figure 5D). Activation of proximal APA was found also for *TAF9* while a proximal 3′ss was promoted in *ATXN7L3*, a component of the deubiquitination module.

### Exon-, isoform- and gene-level control of U2AF35 interaction partners

Over 25% (14/51) genes encoding high-confidence interaction partners of U2AF35 (62) were significantly upregulated or downregulated in depleted cells, which was more than expected (hypergeometric test, *P* < 0.05, Table 1). *U2AF2* showed the highest increase, followed by *CDC5L*, which encodes a key component of the PRPF19-CDC5L complex required for the catalytic step of splicing (63). Upon U2AF35 depletion, the CDC5L interaction partner *PLRG1* showed 3′UTR lengthening but CTNNBL1, CCAP1, CCAP3 and CCAP6 mRNAs were not noticeably altered. SNRPA1, which binds U2 snRNA (64), had reduced mRNA levels in depleted cultures. Transcripts encoding U2AF35-related protein U2AF26, which can interact with U2AF65 and functionally substitute U2AF35 in constitutive and enhancer-dependent splicing (65), were also downregulated while SF3 components *SF3B1* and *SF3B3* showed alterations in APA selection.

**Table 1.** Gene- and exon-level alterations of high-confidence interaction partners of U2AF35 in ab- cells

Partner gene	Gene-level^a^	Exon-level
*U2AF2*	−1.17	NS
*SRSF1*	−0.43	Longer 3′UTR as a result of promotion of distal APA
*SF3B3*	0.54	Longer 3′UTR as a result of promotion of distal APA
*SF3B14*	−0.65	NS
*SF3B1*	NS	Promotion of distal APA, retention of intron containing proximal APA
*CDC5L*	−0.71	NS
*PLRG1*	NS	Longer 3′UTR as a result of promotion of distal APA
*ZCCHC8*	−0.51	NS
*U2AF26*	1.43	NS
*SNRPA1*	0.97	NS
*SAP18*	−0.53	NS
*SON*	−0.58	Promotion of putative proximal APA site
*MCM5*	−0.55	NS
*NHP2L1*	−0.47	Promotion of proximal alternative transcription initiation site

^a^Negative log_2_-fold values indicate upregulation in ab- cells; positive values indicate downregulation. NS, not significantly altered by U2AF35 depletion.

Supplementary Figure S13 shows examples of exon usage dependencies of high-confidence U2AF35 interaction partners. In depleted cells, PTC-containing cryptic exons in *U2AF2* and *CAPERα* were downregulated and both genes were upregulated. Expression of CAPERβ exon 4 was also increased while *PUF60* exon 5 was downregulated. Alternative 5′ss of *U2AF2* exon 10, which controls the inclusion of four amino acids in U2AF65, was not altered by U2AF35 depletion and the two *U2AF2* isoforms were upregulated in depleted cells to the same extent (Figure 1F). Together, these data identify high-confidence interaction partners of U2AF35 whose expression was altered upon U2AF(35) depletion and reveal exon-centric regulation of closely related *U2AF* genes.

### Evidence for a distinct function of U2AF35 isoforms

**Figure 6.**
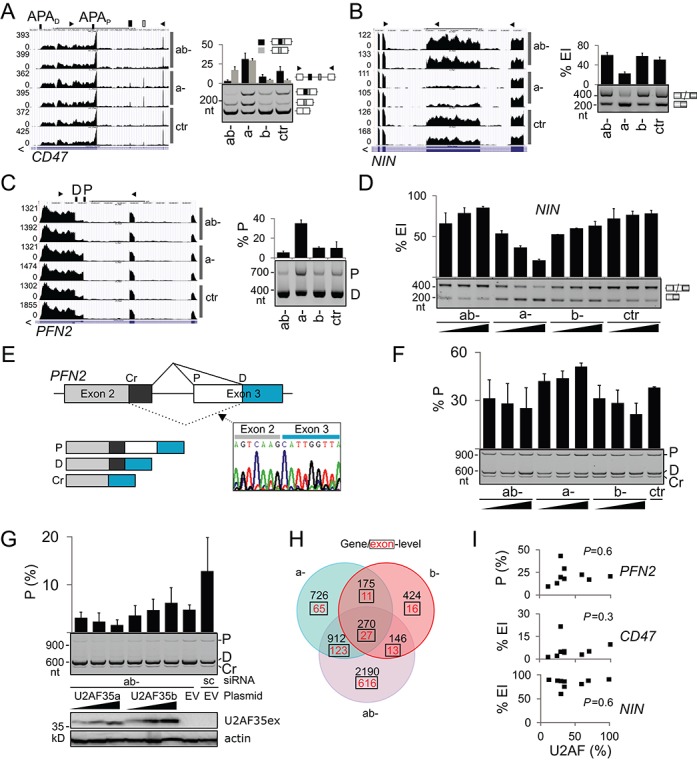
Evidence for distinct function of alternatively spliced U2AF35 isoforms. (**A-C**) Genome browser views of endogenous transcripts showing isoform-specific responses to U2AF35 depletion (left panels) and their validation using RT-PCR (right panels). (**D**) *NIN* exon inclusion levels in the indicated depletions. Final concentration of siRNAs was 6.7, 20 and 60 nM. Error bars are SDs. (**E**) Schematics of the *PFN2* minigene. Chromatogram illustrates transcripts spliced to the cryptic 5′ss of intron 2 (Cr); P, D, proximal and distal 3′ss. (**F**) Opposite effects of U2AF35a and U2AF35b on splice-site selection in exogenous *PFN2* transcripts. Spliced products are shown in panel E. (**G**) Isoform-specific rescue of 3′ss of *PFN2* intron 2. The amount of rescue plasmid DNA was 20, 65 and 200 ng. Immunoblot with Xpress (U2AF35ex) and β-actin antibodies is shown in the lower panel. (**H**) Three-way Venn diagram showing overlaps of differentially expressed genes/exons (q < 0.05) in ab-, a- and b- depletions versus controls. Gene lists are in Supplementary Tables S2 and S3. (**I**) Exon/proximal 3′ss usage in the indicated transcripts (y axis) and residual U2AF heterodimer levels (x axis) estimated from a transfection experiment shown in Supplementary Figure S3.

U2AF35a and b differ from each other at seven amino-acid positions located in the RNP2 motif of the UHM, a proximal part of a long α-helix A and a disordered segment between the two folded regions (13). Isoform-specific depletions identified transcripts exhibiting a gradient in RNA processing defects reflecting total levels of U2AF(35) but also exons activated only upon ab- depletion where U2AF levels were the lowest (Supplementary Figures S2 and S3A,B). However, we also found events that occurred only in b- (Supplementary Figure S14) and a- cultures (Figure 6A–C), in which the less abundant *U2AF1b* was in excess (Figure 1E and Supplementary Figure S3A, lane 6). They were confirmed in independent transfections using varying siRNA concentrations (Figure 6D) and with exogenous transcripts (Figure 6E and F). For example, U2AF35a depletion activated a proximal 3′ss of *PFN2* intron 2 and a distal cryptic 5′ss of the same intron whereas U2AF35b depletion was associated with the opposite effect in a dose-dependent manner (Figure 6F). The proximal 3′ss was promoted by U2AF35b and repressed by U2AF35a also in reconstitution experiments in which we individually added plasmids expressing each isoform to ab- cells (Figure 6G). Repression and activation of the *PFN2* 3′ss was confirmed in cultures depleted with SSOa and SSOb (Supplementary Figure S1B and data not shown). Alternative 3′ss of *PFN2* generate isoforms with distinct C-termini of profilin 2, a key actin-monomer binding protein, that have distinct binding affinities for proline-rich sequences and show tissue-specific expression (66,67). Interestingly, exogenous U2AF35b expression was higher compared to U2AF35a (Figure 6G). The existence of isoform-specific effects was also supported by the number of differentially expressed genes/exons in isoform-specific depletions, with a significant overlap in each category and a low ratio of exon-level versus gene-level events in b- samples (Figure 6H). In contrast to most transcripts, correlation between U2AF levels and exon usage was absent or decreased for genes with isoform-specific responses (cf. Figure 6I and Supplementary Figure S3).

To reinforce these findings further, we carried out independent isoform-specific depletion experiments with total RNA depleted of rRNA. These samples contain a higher fraction of unprocessed RNA than polyA-selected RNA, giving more information about intron splicing (68). DEXSeq analysis followed by browser-assisted verification revealed that the bias toward start and terminal exons was even greater than for poly(A) samples and reconfirmed isoform-specific effects validated experimentally (Supplementary Table S5 and Figure 6 and Supplementary Figure S14).

Taken together, identification of transcripts with distinct responses to U2AF35a and U2AF35b argues for the existence of isoform-specific interactions that may confer even opposite effects on splice-site selection and provide additional level of exon regulation.

## DISCUSSION

We have shown the first global characterization of RNA processing alterations associated with depletion of U2AF35 isoforms. Our data reveal that U2AF function is not limited to 3′ss recognition but involves extensive APA control (Figure 2), particularly through intronic APA sites, suggesting that U2AF contributes to the tight regulation of tissue-specific expression. We have also described characteristic 3′ss organization of U2AF(35)-repressed and -activated exons and functional antagonism and synergism of U2AF-related proteins PUF60 and CAPERα (Figure 3). The exon repression and activation was associated with significant shifts of average unpaired probabilities in their canonical BP and PPT regions and further upstream. Finally, we describe exon-centric regulations of genes encoding U2AF35 interaction partners and identify transcripts with distinct responses to U2AF35a and U2AF35b.

Our results indicate that most but not all changes in APA and exon usage in cells depleted of U2AF35 were attributable to the lack of U2AF heterodimer (Supplementary Figure S3 and Figure 6I). They were replicated in cells lacking U2AF65 (Figure 3H, Supplementary Figures S2 and S11), in agreement with RASL-Seq data submitted during review of this manuscript and showing significant overlap of U2AF35- and U2AF65-induced events (69). As U2AF65 interacts with the 3′ end processing complex, the association of U2AF with the phosphorylated C-terminal domain (CTD) of polII and PRPF19 (70,71) could be important for the U2AF-dependent APA control. U2AF35 depletion was associated with upregulation of *CDC5L* (Table 1), a key component of the PRPF19-CDC5L complex required for promotion of co-transcriptional splicing (72). The impaired balance between post- and co-transcriptional splicing in cells lacking U2AF is supported by frequent alterations of weak introns that contained cryptic or alternative exons, often near 3′ gene ends (Supplementary Figures S5C and S13). These ‘detained’ introns are spliced post-transcriptionally in humans (73). In *Drosophila* and the mouse, alternative introns are less efficiently spliced co-transcriptionally than constitutive introns and co-transcriptional splicing is less efficient toward 3′ gene ends than at upstream gene locations (74). The link between U2AF and transcription and polII elongation rate (75), which can alter APA choice (76), is further supported by a biased distribution of differentially used start and terminal exons in cells depleted of elongation factors (Supplementary Table S5) and significant overlap of genes differentially expressed in U2AF35-, hnRNP C- and AFF4-depleted cells (Supplementary Figure S15). AFF4 is a key elongation factor while hnRNP C directly competes with U2AF65 at authentic and cryptic 3′ss (25), in agreement with the observed tendency to antagonize U2AF(35)-repressed exons (Figure 3H). Altered elongation rates influenced inclusion of ∼15–40% cassette exons on a genome-wide scale and the slow elongation has been associated with shorter introns (77), which were prevalent among U2AF-sensitive events (Supplementary Figure S7).

The APA selection was influenced by U2AF in an APA category-dependent manner (Figure 2C–E). U2AF appears to activate proximal APA sites if there is no intron between competing APA sites and distal APA sites if recognition of the 5′ss is productive (Figure 2C and D). U2AF65 contacts CFIm59 but not CFIm68 (78), which promoted distal APA sites (79). PolII CTD also interacts with the CFIm subunit PCF11 (80) and several splicing factors, including PRP40, CA150 and PSF (81–83). Significant shifts in cleavage site usage, largely toward proximal sites, were observed for a knockdown of PABPN1 (84), a nuclear protein with high affinity to poly(A) tails, with 43 genes shared between PABPN1- and U2AF35-depleted samples (*P* < 10^−8^, data not shown). Finally, U2AF-dependent intronic APA sites represented the most frequent APA category (Figure 2E), whereas in normal cells intronic APA sites are less frequent than tandem 3′UTR and alternative terminal exons (85), which could be due to widespread binding of U2AF(65) to introns, with >80% tags in intronic sequences (69).

Similar to other RNA-binding proteins (50,51,86,87), U2AF(35) can both inhibit and activate splicing (Supplementary Figures S2 and S3). Activated and repressed exons had a distinct 3′ss organization and functional regulation by Y-binding proteins (Figure 3, Supplementary Figures S10–S12). The U2AF(35)-repressed exons were largely stimulated by PUF60 (Supplementary Figure S11A) and inhibited by CAPERα and also by PTB, in line with longer AGEZs/PPTs previously found for PTB-repressed cassettes (50). Because PUF60 preferentially contacts uridines (88), the PUF60-U2AF antagonism might be explained by competition for binding to uridine-rich sequences. However, uridine was enriched upstream of both upregulated and downregulated exons (Supplementary Figure S10) and also within these exons (Supplementary Figure S7B), arguing for the importance of adenine/uridine frequency shifts between optimally located BP and PPT regions (Supplementary Figure S10A) and between regions showing higher single-strandedness (Figure 3J). These changes are likely to alter not only protein binding but also tertiary contacts and folding transitions by helicases and other RNA chaperones. Structural requirements across 3′ss may also help explain atypical responses of some transcripts, such as *GSK3B* to PUF60 depletion (Supplementary Figure S11A), and position-dependent alternative splicing activity of a growing number of proteins and their targets (50,89–91).

PUF60 contains two RNA recognition motifs (RRMs) and a C-terminal UHM (88). This UHM binds the N-terminus of SF3b155 at the UHM-ligand motif (ULM) around W200 (92). The functional PUF60-U2AF antagonism might also result from competition of U2AF35 and PUF60 for the U2AF65 ULM because binding of PUF60 to the U2AF65 ULM can only occur if this motif is not already bound by the U2AF35 UHM (92). In contrast, PUF60 and U2AF65 can bind to the N terminus of SF3b155 simultaneously and noncompetitively (92). In addition, U2AF and PUF60 had the opposite effect on BP accessibility and U2AF was not strictly required for splicing of some pre-mRNAs *in vitro* when PUF60 was present (51). Finally, anti-PUF60 antibodies co-precipitated polII CTD and three components of the general transcription factor TFIIH (93), linking PUF60 to transcription. Unlike PUF60 on U2AF-repressed exons (Figure 3H), however, slow and fast polII elongation do not usually have opposite effects on the inclusion of a given alternative exon (77).

Our study revealed activation and inhibition of many cryptic exons, both in U2AF-related and unrelated genes. The elevated *U2AF2* mRNA (Figure 1D) can be explained by repression of a PTC-containing exon in intron 5 in depleted cells (Supplementary Figure S13). This exon is highly conserved between mouse and man, has a GC 5′ss and is surrounded by Y-rich sequences. Such cryptic exon activation appeared to be common in other U2AF35 partners (Supplementary Figure S13, Table 1). Similar to *U2AF2, CAPERα* was upregulated upon U2AF depletion, most likely through elimination of a PTC-introducing cryptic exon (Supplementary Figure S13). CAPERα showed a strong synergism with U2AF on upregulated exons (Figure 3H) and has the same domain structure as U2AF65, except for the lack of ULM (94). In contrast, a 48-nt *CAPERβ* exon activated in ab- cells (Supplementary Figure S13) does not present PTCs but is translated only if upstream alternative exons are included in the mRNA. The mRNA expression of PUF60, which lacks both the ULM and the RS domain (94), was unaffected, although *PUF60* exon 5 appeared to respond positively to the excess of U2AF35b (Supplementary Figure S13). This 51-nt cassette exon introduces extra 17 amino acids close to the first RRM of PUF60 and encodes two serines that are phosphorylated (95).

Although positions −3 and +1 relative to U2AF(35)-dependent 3′ss may participate in U2AF35-RNA interactions, with binding preferences favoring cytosine at position −3 and guanine at position +1 (Supplementary Figure S4), possibly via ZFs (24), experimental evidence for this interaction is missing. The higher dependency of weaker and proximal alternative 3′ss on U2AF35 was finally seen at the genome-wide level (Supplementary Figure S4, Table S9), resolving previous uncertainties (14,24). However, U2AF(35)-dependent exons and 3′ss were largely alternatively spliced, which makes it difficult to distinguish characteristic sequence features of cassette exons from direct effects of U2AF35 depletion. For example, alternative splicing and exon skipping was found to correlate positively with the BP-3′ss distance (41). The distance between 3′ss and the best predicted BP of exons/3′ss upregulated in ab- cells was marginally higher than in controls and these 3′ss had also a higher number of BPs with positive SVM scores (data not shown). Thus, it remains to be tested how BP choice is affected by a lack of U2AF(35) and by the observed positional changes in single-strandedness (Figure 3J). Interestingly, cooperative interactions of U2AF and SF1 increased the SF1 binding repertoire and SF1 binding was significantly biased toward terminal exons (87).

The higher exogenous expression of U2AF35b over U2AF35a (Figure 6G) and elevated relative abundance of U2AF35b upon U2AF65 depletion (Supplementary Figure S3) suggest that interactions of each isoform with U2AF65 could be important for U2AF stability, thus contributing to tight regulation of U2AF levels *in vivo* and accurate exon/APA usage. Description of exons with isoform-specific responses to U2AF35 should facilitate characterization of physical interactions of highly conserved U2AF35a and U2AF35b isoforms in future studies. These interactions may involve the extraordinarily long α helix A (96) and are likely to be affected by post-translational modifications of residues encoded by exon 3/Ab as the size difference between U2AF35b and U2AF35a appears to be larger than predicted (Figure 1C and Supplementary Figure S1).

Although U2AF35 is believed to be a 3′ss recognition factor, we found many examples of 5′ss usage alterations upon depletion (Supplementary Figure S5). This may be explained by altered U2AF65-promoted recruitment of U1 snRNP to weak 5′ss (97) but also elongation kinetics of polII. For example, Rsd1, a yeast homologue of CAPERα, bridges interactions between U1 and U2 snRNPs through the RS domain of Prp5 (DDX46) (98), which was repressed in ab- cultures (Supplementary Table S2). Prp5 is required for a transcription elongation checkpoint and a release of stalled polII (99). Differentially used alternative 5′ss were also found in cells lacking other RNA-binding proteins, including PTB (50), nevertheless a large excess of U2AF(35)-dependent 3′ss over 5′ss in our data set is consistent with the predominant role of this factor in 3′ss recognition.

Finally, genome-wide identification of U2AF(35)-dependent events and our validation panel will provide an important resource for more detailed biochemical and structural studies of 3′ss and APA sites, expanding not only the number of U2AF35-dependent exons but also exons sensitive to other factors involved in 3′ss/BP/APA selection.

## ACCESSION NUMBER

RNA-Seq data are available at ArrayExpress (E-MTAB-2682).

## SUPPLEMENTARY DATA

Supplementary Data are available at NAR Online.

SUPPLEMENTARY DATA
