# Supplementary material for: Identification of U2AF(35)-dependent exons by RNA-Seq reveals a link between 3′ splice-site organization and activity of U2AF-related proteins
Source: Nucleic Acids Res. 2015 Mar 16;43(7):3747–63. doi: 10.1093/nar/gkv194 (PMC4402522; doi:10.1093/nar/gkv194)
Supplement: SUPPLEMENTARY DATA [file supp_43_7_3747__index.html]

Identification of U2AF(35)-dependent exons by RNA-Seq reveals a link between 3′ splice-site organization and activity of U2AF-related proteins — Identification of U2AF(35)-dependent exons by RNA-Seq reveals a link between 3′ splice-site organization and activity of U2AF-related proteins — SUPPLEMENTARY DATA 

# Identification of U2AF(35)-dependent exons by RNA-Seq reveals a link between 3′ splice-site organization and activity of U2AF-related proteins

## SUPPLEMENTARY DATA

**Files in this Data Supplement:**

- SUPPLEMENTARY DATA
- SUPPLEMENTARY DATA
