# Supplementary material for: Identification of U2AF(35)-dependent exons by RNA-Seq reveals a link between 3′ splice-site organization and activity of U2AF-related proteins
Source: Nucleic Acids Res. 2015 Mar 16;43(7):3747–63. doi: 10.1093/nar/gkv194 (PMC4402522; doi:10.1093/nar/gkv194)
Supplement: SUPPLEMENTARY DATA [file supp_gkv194_nar-00200-a-2015-File003.pdf]

SUPPLEMENTARY FIGURES

Identification of U2AF(35)-dependent exons by RNA-Seq reveals a link between 3' splice-site organization and activity of U2AF-related proteins

Jana Kralovicova[1], Marcin Knut[1], Nicholas C. P. Cross[1,2], Igor Vorechovsky[1]

[1]University of Southampton
Faculty of Medicine
Southampton SO16 6YD
United Kingdom

[2]Wessex Regional Genetics Laboratory
Salisbury District Hospital
Salisbury SP2 8BJ
United Kingdom

Figure S1 Isoform-specific knockdown of U2AF35

A,B, Time-course transfection experiment with splice-switching oligonucleotides (SSOs) and siRNAs targeting U2AF35*a* and U2AF35*b* isoforms (*top*). Time between the first hit and cell lysis is shown to the left; c, control. **A,** Immunoblots with antibodies against U2AF35 and β-actin. Final concentrations of SSOs and siRNAs were 60 nM. siRNAs were described previously (1-3); sequences of SSOs are in Table S4. **B,** *U2AF1* splice products detected by RT-PCR; b/a, ratio of *U1AF1b* and *U2AF1a*; c, inclusion of both alternative exons; s, skipping of both exons. Error bars denote SDs of duplicate transfections.

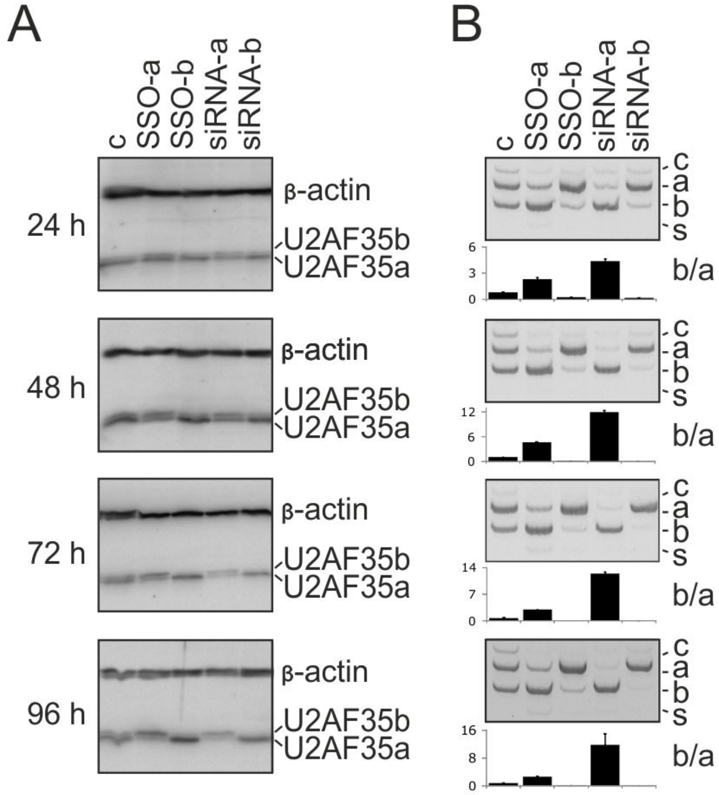

Figure S2 Validation of U2AF(35)-dependent exons by RT-PCR

Examples of RNA processing defects detected by DEXSeq in cultures depleted of U2AF35 and its isoforms (**A**) and of U2AF65 (**B**). Gene symbols are shown to the left, RNA products to the right. EI, exon inclusion, ES, exon skipping. Error bars are SDs of two replicates. PCR primers are in Table S4. Corresponding immunoblots are shown in Figs. 1C and 2I and FPKMs in Figs. 1D-F.

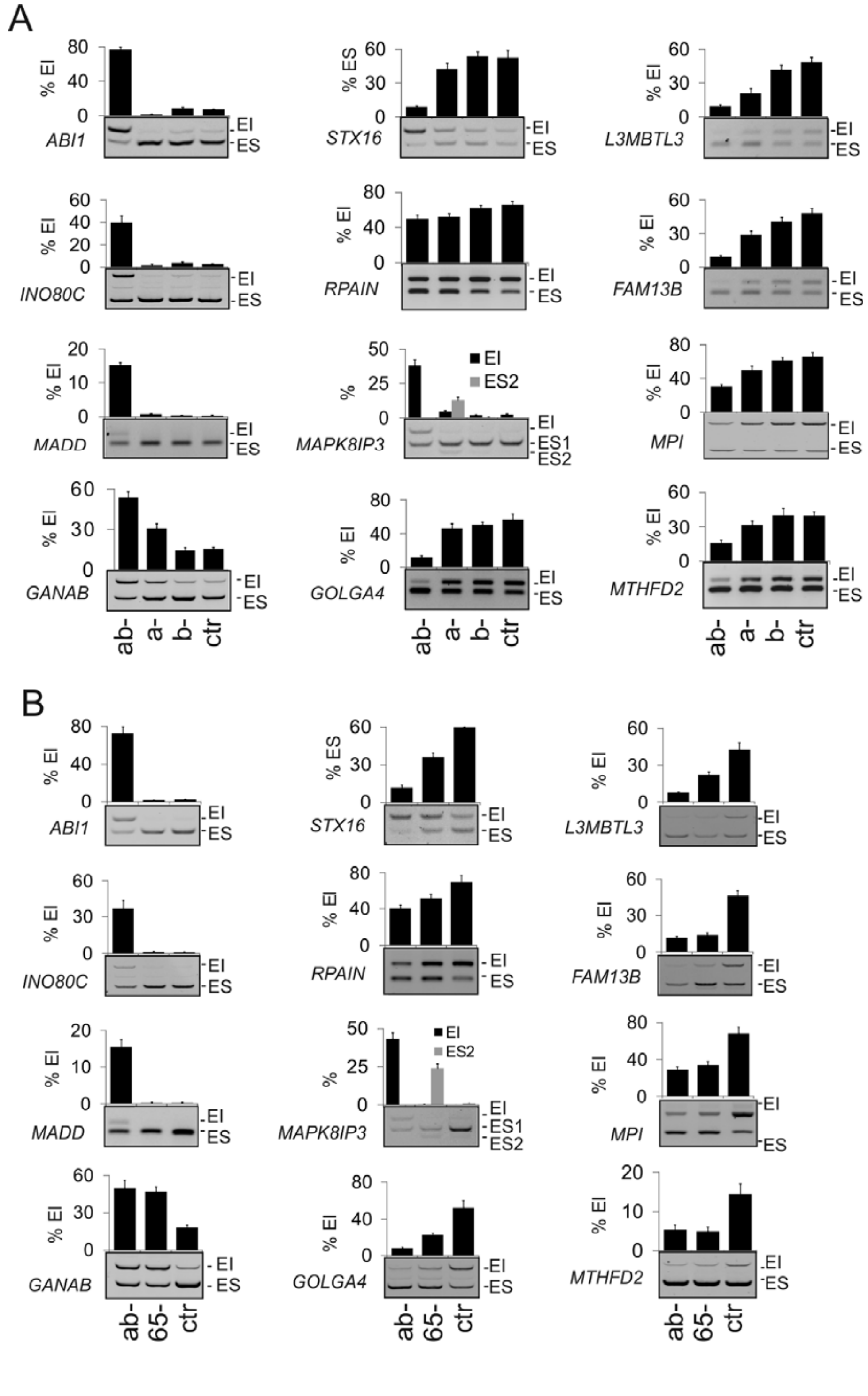

Figure S3 Correlation of U2AF levels and exon usage

A, Immunoblots of a transfection experiment with the indicated siRNAs (*upper panel*) and *Hinf*I-digested RT-PCR of *U2AF1* transcripts (*lower panel*). A final concentration of U2AF35ab- siRNA was 3, 10 and 30 nM. A final concentration of U2AF65 siRNA was 30 and 50 nM. Isoform-specific siRNAs were added to a final concentration of 50 nM. Lane 2 contains 25% of the control lysate/PCR product. **B,** U2AF(35)-dependent transcripts (*left panel*) and correlation between U2AF heterodimer levels and exon inclusion (EI) or skipping (ES) (*right panel*). ES1/ES2, skipping of one/two exons. P-values for Pearson correlation coefficients were derived from t-tests. **C,** Transcripts with isoform-specific responses amplified by RT-PCR from the same transfection experiment. Correlation of their exon usage with U2AF is shown in Fig. 6I. **D,** Summary of *MAPK8IP3* exon usage. The size of RNA products is to the left. AGEZ, AG exclusion zone.

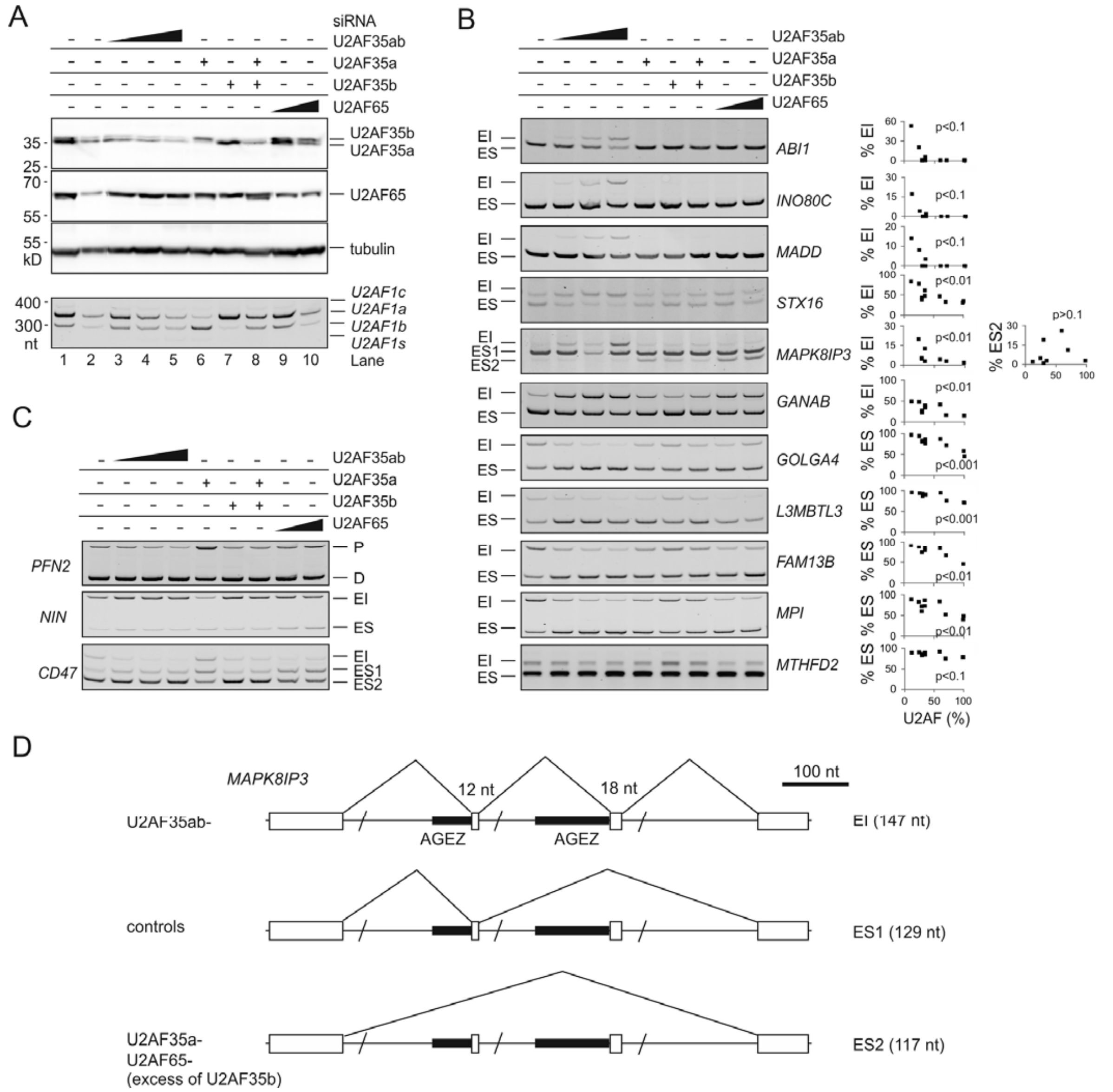

Figure S4 Nucleotides at position -3 and -1 relative to U2AF(35)-dependent alternative 3' splice sites

Number of 3'ss with the indicated nucleotides at position -3 **(A)** and position +1 **(B).** The total number of upregulated proximal 3'ss was 93. The total number of upregulated distal 3'ss was 45. P values were derived from χ^2 tests for 4x2 contingency tables.

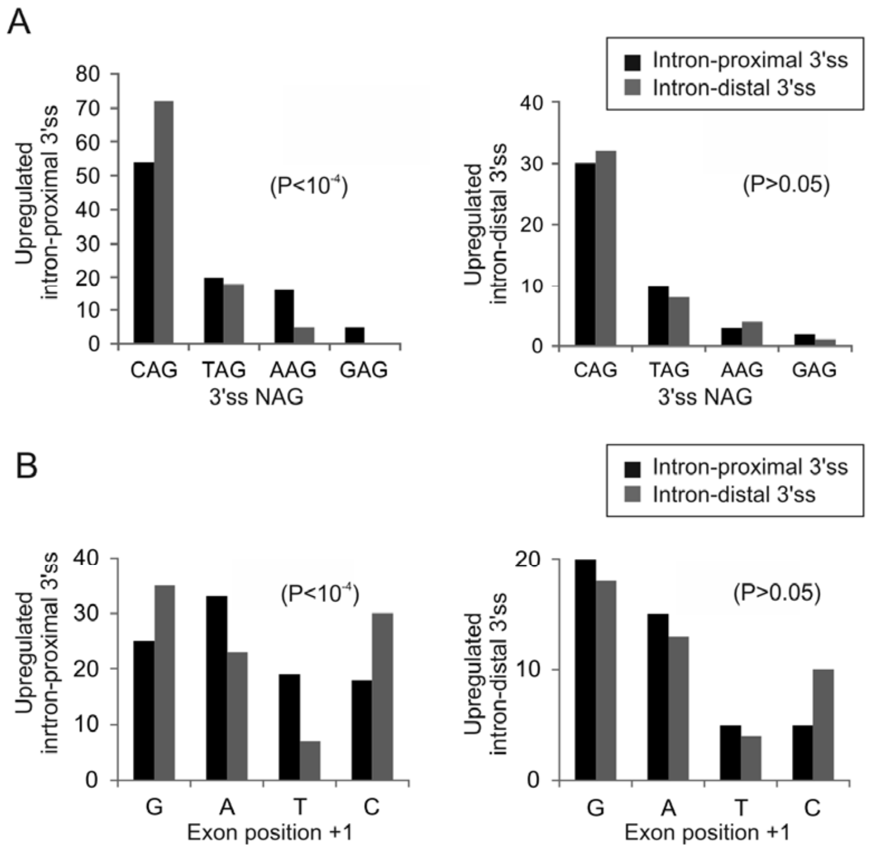

Figure S5 Activation and repression of alternative 5' splice sites in depleted cultures

A, *FUZ,* **B,** *FDXR*, **C,** *HMGN3,* **D,** *CRK,* **E,** *cXorf57,* **F,** *KAT2A,* **G,** *KAT2B,* **H,** *MPDU1.* P, proximal 5'ss, D, distal 5'ss. Genome browser views of RNA-Seq data from ab- and control cultures are shown to the left. Primers (Table S4) are denoted by arrowheads. Depletions are indicated at the bottom. Exonic segments up-/down-regulated in depleted cells are denoted by red/green rectangles.

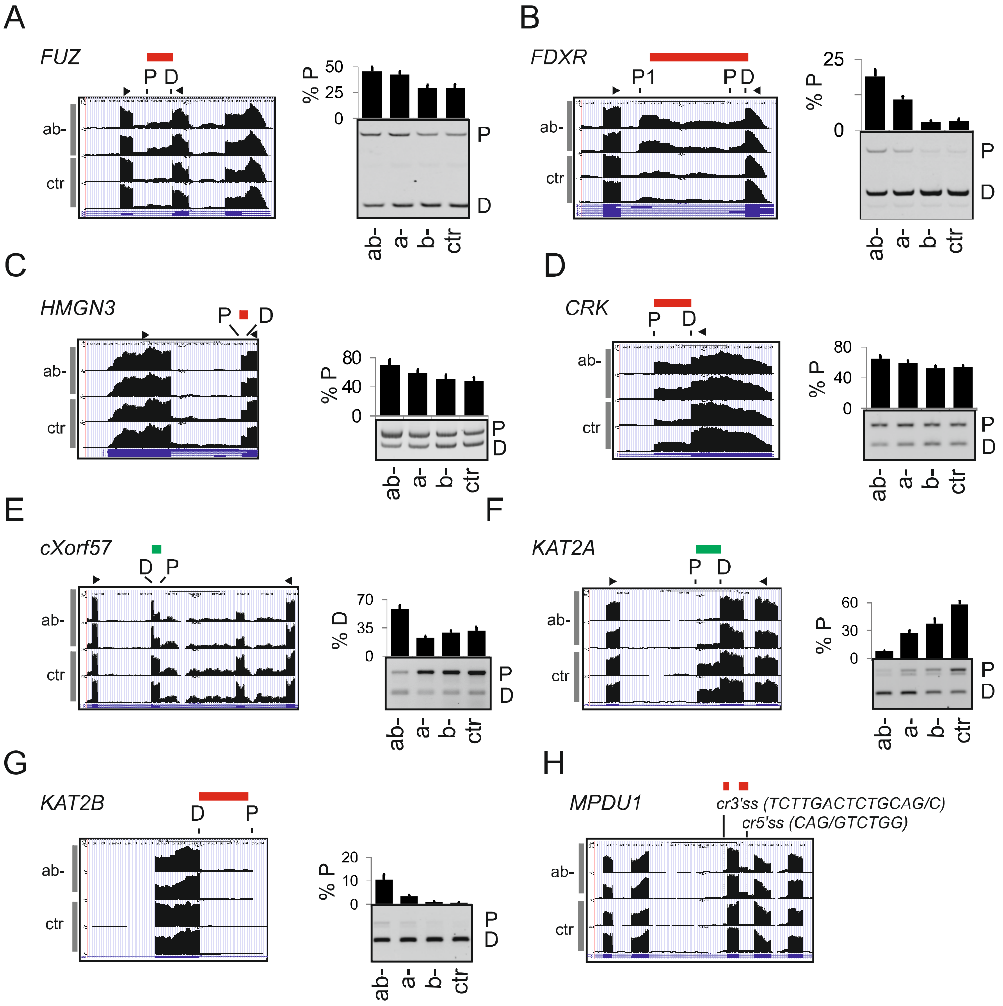

A
FUZ
P D
ab-
ctr
% P
50
25
0
P
D
ab-
a-
b-
ctr
B
FDXR
P1
P D
ab-
ctr
% P
25
0
P
D
ab-
a-
b-
ctr
C
HMGN3
P D
ab-
ctr
% P
80
40
0
P
D
ab-
a-
b-
ctr
D
CRK
P D
ab-
ctr
% P
80
40
0
P
D
ab-
a-
b-
ctr
E
cXorf57
D P
ab-
ctr
% D
70
35
0
P
D
ab-
a-
b-
ctr
F
KAT2A
P D
ab-
ctr
% P
60
30
0
P
D
ab-
a-
b-
ctr
G
KAT2B
D P
ab-
ctr
% P
20
10
0
P
D
ab-
a-
b-
ctr
H
MPDU1
cr3'ss (TCTTGACTCTGCAG/C)
cr5'ss (CAG/GTCTGG)
ab-
ctr

Figure S6 U2AF(35) depletion can promote intron splicing and exon inclusion

A, Reduction of intron retention upon U2AF35 depletion. DEXSeq-detected downregulation of a 3'UTR intron (green rectangle), resulting in a shorter 3'UTR, which was associated with *VAMP1* upregulation (Table S3). **B,** Intronization of a mid-portion of *C2orf49* exon 3 (green rectangle), employing a GC 5'ss (CAG/GCAAGC, where / is the exon-intron junction). **C,** Examples of upregulated exons (red rectangles) within intronic sequences incompletely eliminated from the indicated pre-mRNAs in untreated cells.

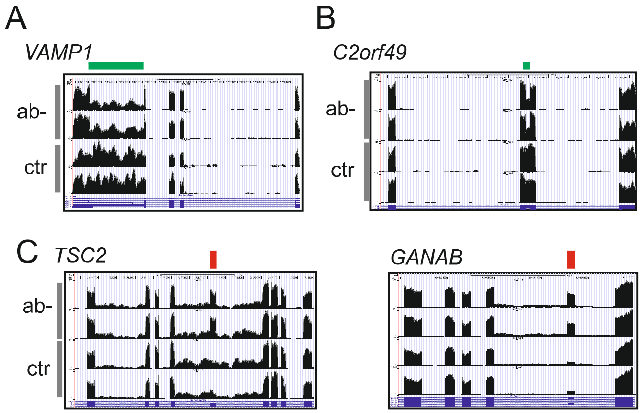
A
VAMP1
ab-
ctr
B
C2orf49
ab-
ctr
C
TSC2
ab-
ctr
GANAB

Figure S7 U2AF(35) –dependent exons are smaller and are depleted of guanine and enriched in uridine as compared to average human exons

A, Size distribution of U2AF(35)-dependent internal exons. Median is denoted by a dotted vertical line. Size distribution of DEXSeq-detected pre-mRNA segments annotated as introns is shown in the inset. **B,** Nucleotide frequencies in differentially used internal exons. ****, P<0.00005; NS, not significant. **C,** Nucleotide frequencies at position +1 of U2AF(35)-dependent exons. *, P<0.05. The first position of U2AF35-dependent exons was also depleted of guanine and showed the same hierarchy in splicing efficiency (G>A>C>T) as a distal cryptic 3'ss induced by a lack of U2AF35 or overexpression of U2AF65 (3). Organization of U2AF(35)-dependent 3'ss is shown in Fig. 3 and Figs. S8-S10.

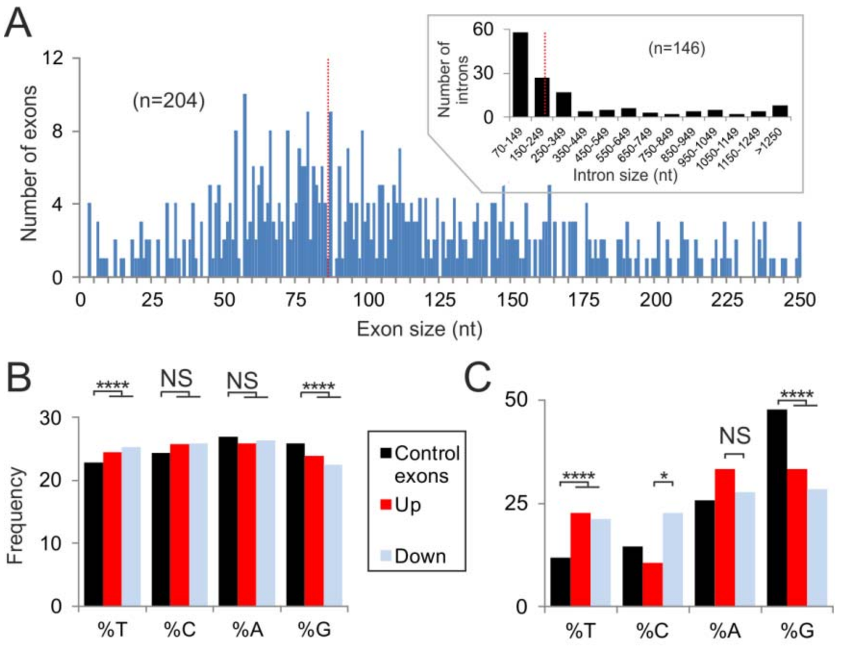

Figures S8 Lack of AG dinucleotides in the last 50 nt of introns upstream of internal exons (A) and differentially used alternative 3' splice sites (B) in cells depleted of U2AF35.

Columns represent means, error bars denote SDs. P-values were derived from two-tail t-tests. The number of up- and down-regulated internal exons was 67 and 137, respectively. The number of alternative 3'ss pairs was 138. The number of control exons was 177,290.

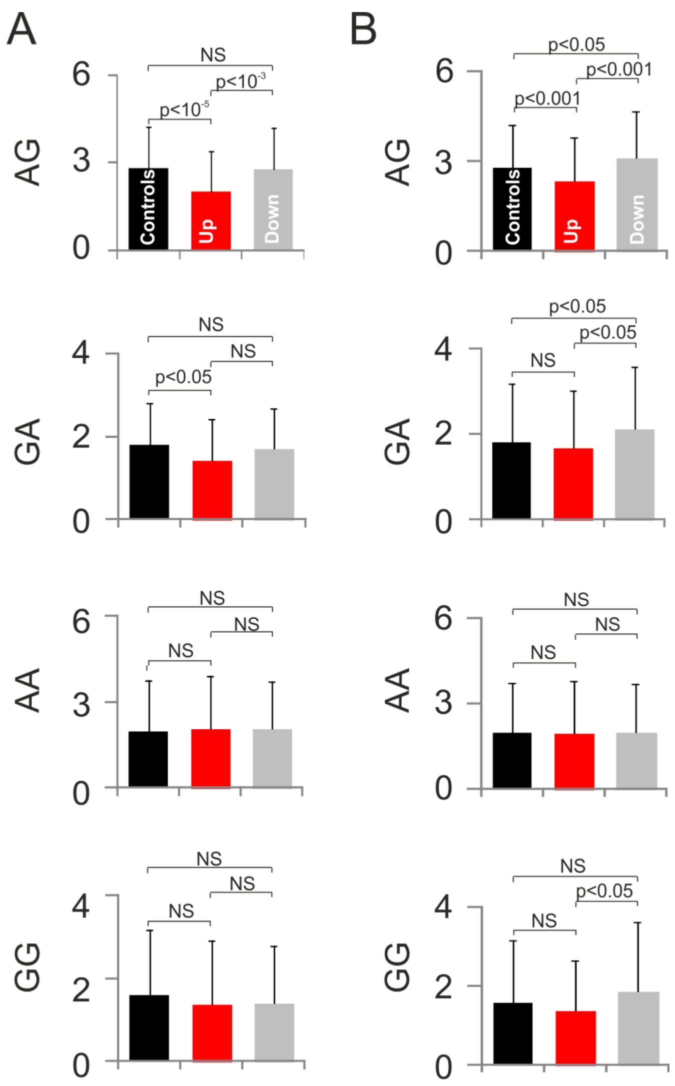

Figure S9 AGEZ/PPT length of alternative 3'ss leading to differentially used APA sites

A, AGEZ length, **B,** PPT length. Each APA 3'ss (n=57, Fig. 2E) is shown in Table S6. Error bars are SDs; P-values were derived from t-tests.

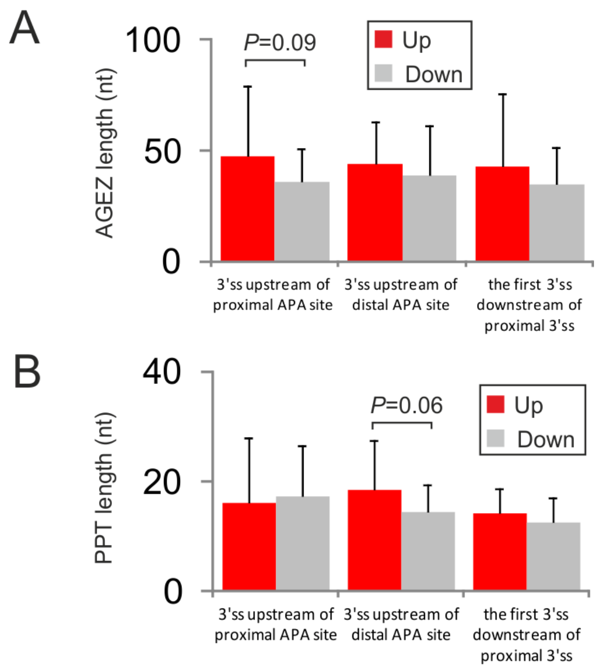

Figure S10 Organization of 3' splice sites of U2AF(35)-dependent exons

A, Nucleotide frequencies upstream of 3'ss of internal exons up-/down-regulated in cells depleted of U2AF35 (n=204) and control exons (n=177,290). Depletion/excess of adenine/uridine in upregulated exons in the canonical BP (red rectangle) and PPT (black rectangle) locations is highlighted; asterisks denote positions exhibiting significant differences (P<0.05) in adenine frequencies between up- and down-regulated exons in these regions. **B,** Systematic comparisons of nucleotide frequencies in 50-nt sequences upstream of U2AF(35)-sensitive internal exons. Error bars denote SDs. *, P<0.05, **, P<0.005, ***, P<0.0005, ****, P<0.00005; t-tests. **C-E,** The same frequencies by size of exons up- **(C)** and down- **(D)** regulated in ab- cultures and control exons **(E)**. Number of analyzed exons is shown to the right.

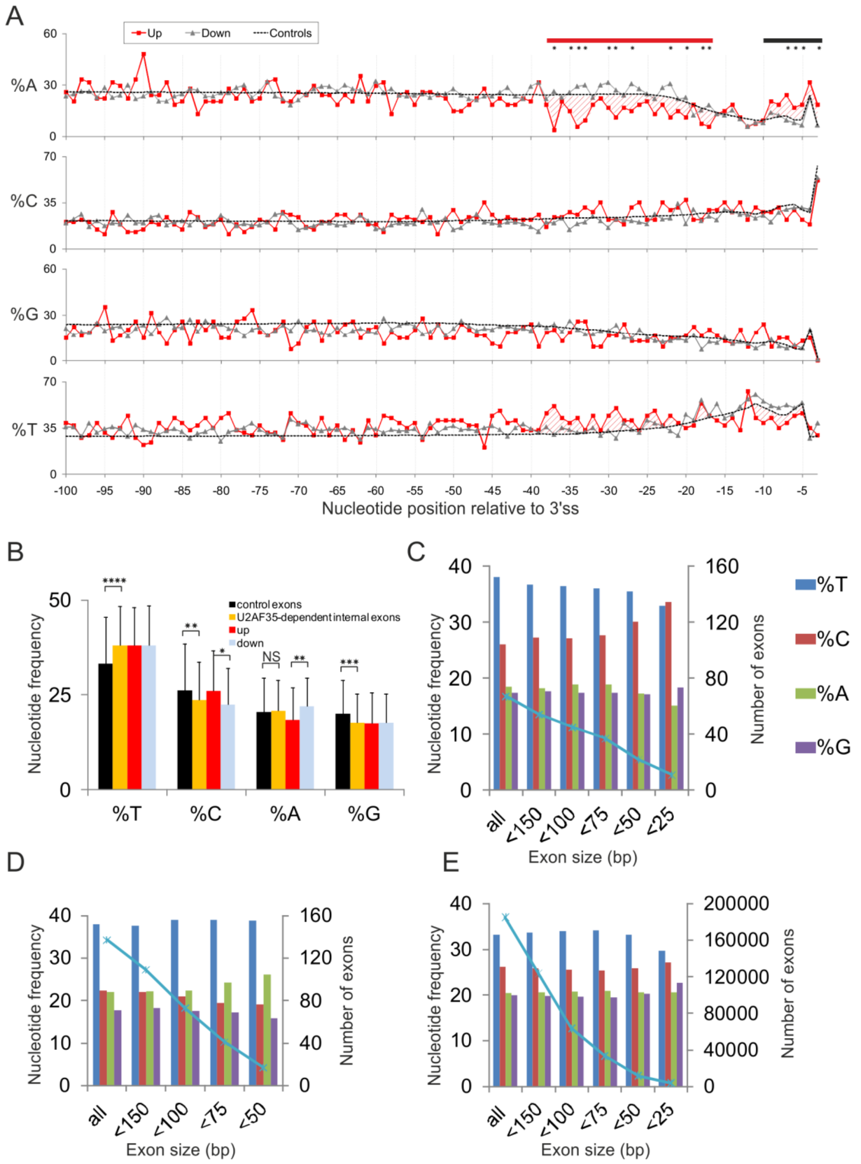

Figure S11 Antagonism and synergism of U2AF-related proteins in U2AF(35)-dependent exons

A, Exons upregulated in cells depleted of U2AF35. **B,** Exons downregulated in cells depleted of U2AF35. **C,** Exons with isoform-specific responses. ES, exon skipping; EI, exon inclusion; P, D, proximal and distal 3'ss; cr, cryptic splice-site. Lane 1, size marker. Columns represent mean %EI (panels A and C) or %ES (panel B); error bars are SDs of two transfections into HEK293 cells. Amplifications primers are in Table S4. Pre-mRNAs containing >3 exons are schematically shown at the top. Immunoblots are in Fig. 3G and estimates of residual protein levels in Fig. 3H. Depletion levels of CAPERβ were estimated only at the RNA level (data not shown).

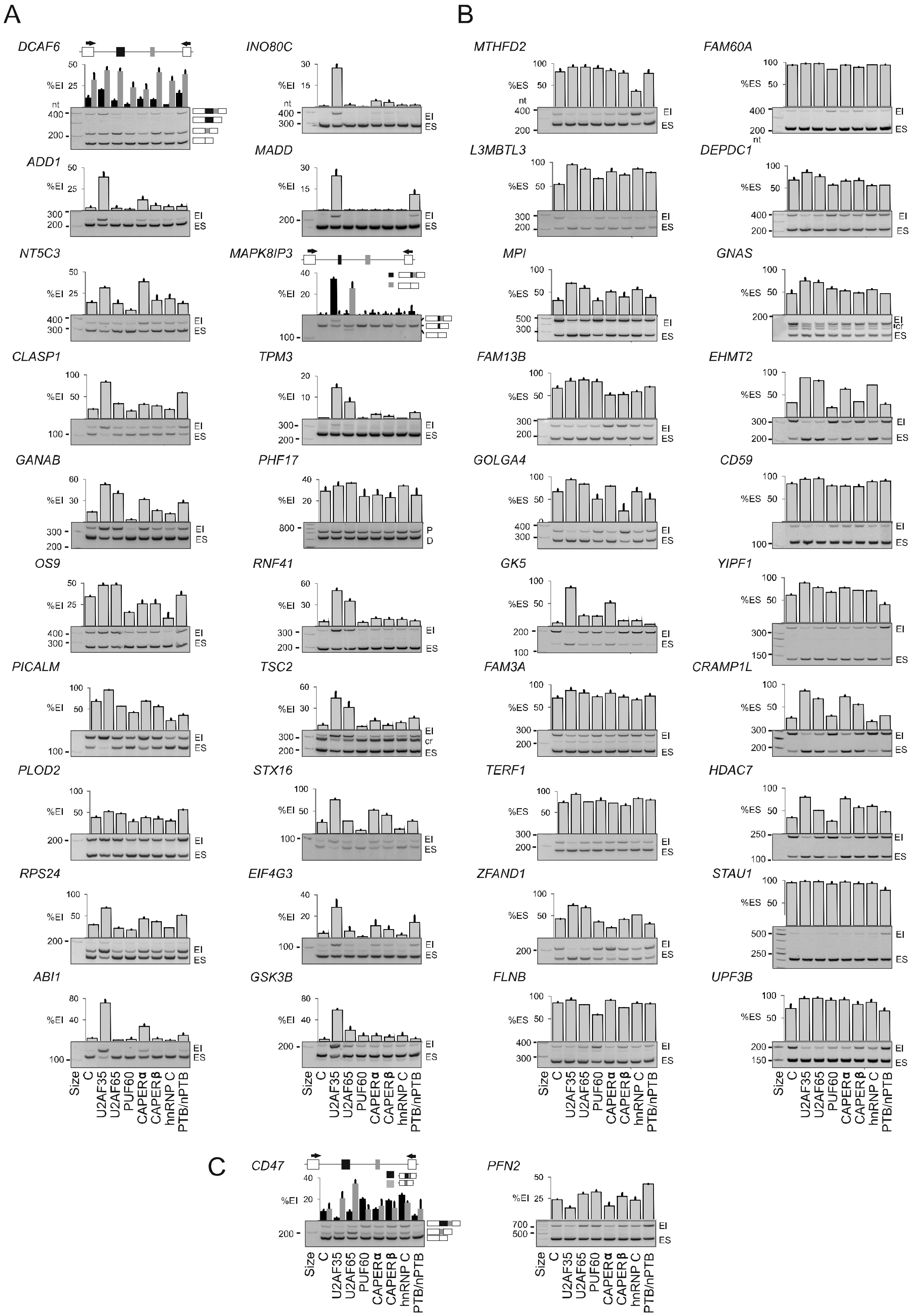
A
B
C
DCAF6
INO80C
ADD1
MADD
NT5C3
MAPK8IP3
CLASP1
TPM3
GANAB
PHF17
OS9
RNF41
PICALM
TSC2
PLOD2
STX16
RPS24
EIF4G3
ABI1
GSK3B
MTHFD2
FAM60A
L3MBTL3
DEPDC1
MPI
GNAS
FAM13B
EHMT2
GOLGA4
CD59
GK5
YIPF1
FAM3A
CRAMP1L
TERF1
HDAC7
ZFAND1
STAU1
FLNB
UPF3B
CD47
PFN2
%EI
%ES
nt
EI
ES
cr
P
D
Size
C
U2AF35
U2AF65
PUF60
CAPERα
CAPERβ
hnRNP C
PTB/nPTB

Figure S12 Tissue-specificity of alternative 3'-end processing of human *SF1*

Distal and proximal APA (APA_D and APA_P) site usage is shown for 3'-seq data described previously (4). The proximal APA site is preferred in B cells, skeletal muscles and embryonic stem cells (hES).

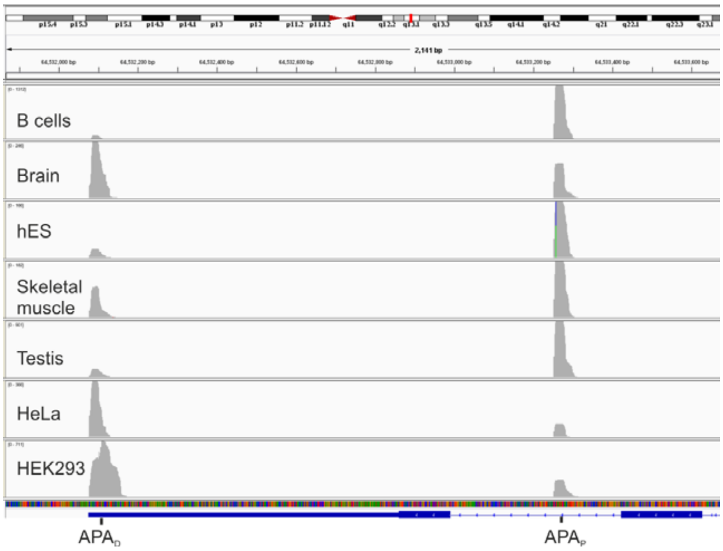

Figure S13 Exon usage dependencies of U2AF35 binding partners

U2AF35 depletion (ab-) was associated with downregulation of a 60-bp cryptic exon in *U2AF2* intron 5, *RBM39* (CAPERα) exon 3, and *PUF60* exon 5, and with upregulation of *RBM23* (CAPERβ) exon 4; a- and b- are isoform-specific depletions of U2AF35. Exon numbers are at the top; vertical arrows denote direction of gene-level expression changes expressed as read numbers (y-axis). Genomic coordinates are at the top and transcript annotations at the bottom (visible after magnification). A functional alternative 3'ss of *RBM39* exon 13 was U2AF(35)-insensitive (data not shown).

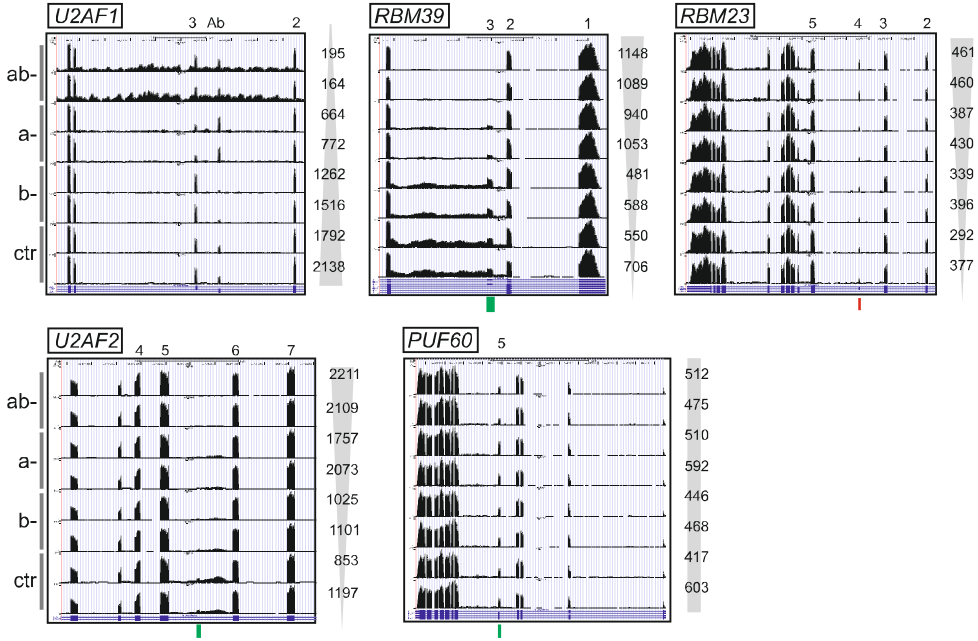

U2AF1
3 Ab 2
195
164
664
772
1262
1516
1792
2138
ab-
a-
b-
ctr
RBM39
3 2 1
1148
1089
940
1053
481
588
550
706
RBM23
5 4 3 2
461
460
387
430
339
396
292
377
U2AF2
4 5 6 7
2211
2109
1757
2073
1025
1101
853
1197
PUF60
5
512
475
510
592
446
468
417
603

Figure S14 Putative *U2AF1b*-specific interactions in regulation of *LAMP2* APA

A, Genome browser view of alternative RNA processing of *LAMP2* transcripts. Annotated APA sites (4) are shown as vertical bars; primers (Table S4) are denoted by arrowheads. mRNA isoforms (*top*) encode distinct C-termini; their peptide sequences are in panel B. **C**, RT-PCR validation showing the relative abundance of isoform B in depleted cells and controls. **D,** Relative abundance of *LAMP2* isoforms in the indicated human tissues. Primers shown to the right correspond to arrowheads in panel A; box denotes multiplex reactions run for 28 cycles; isoform C was amplified in separate reactions for 34 cycles.

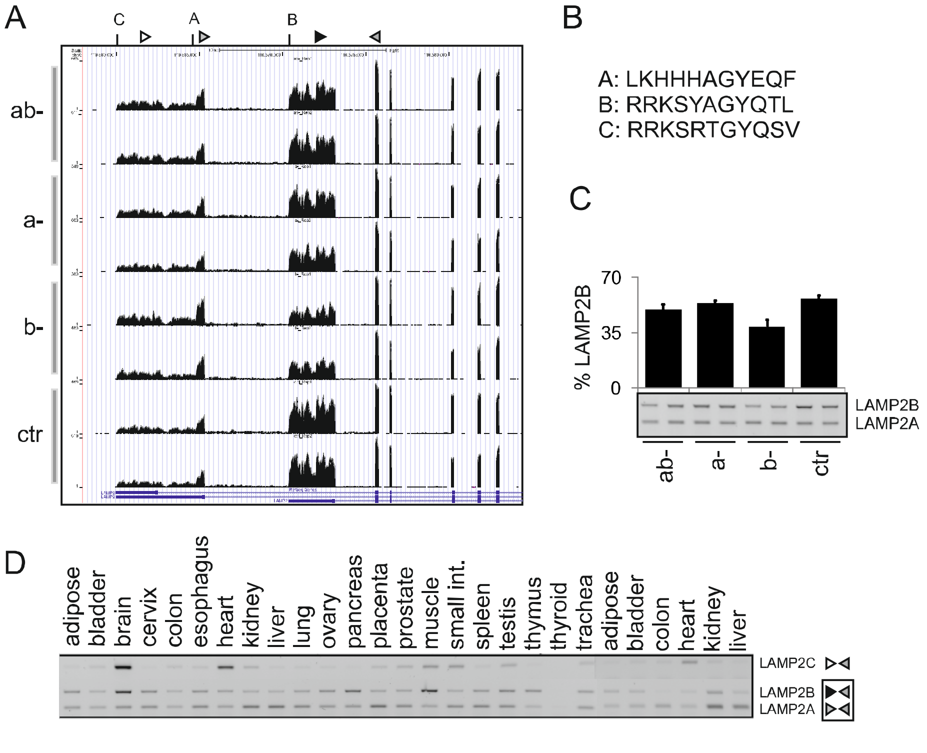
A
C
A
B
ab-
a-
b-
ctr
B
A: LKHHHAGYEQF
B: RRKSYAGYQTL
C: RRKSRTGYQSV
C
70
35
0
% LAMP2B
LAMP2B
LAMP2A
ab-
a-
b-
ctr
D
adipose
bladder
brain
cervix
colon
esophagus
heart
kidney
liver
lung
ovary
pancreas
placenta
prostate
muscle
small int.
spleen
testis
thymus
thyroid
trachea
adipose
bladder
colon
heart
kidney
liver
LAMP2C
LAMP2B
LAMP2A

Figure S15 Transcripts upregulated upon U2AF35 depletion tend to be downregulated in AFF4-depleted cells and vice versa

P-values were derived from hypergeometric and χ^2-tests for the indicated Venn diagrams and contingency tables, respectively. RNA-Seq data were analyzed using Cufflinks from this (U2AF35) and previous studies (hnRNP C, AFF4) (5,6).

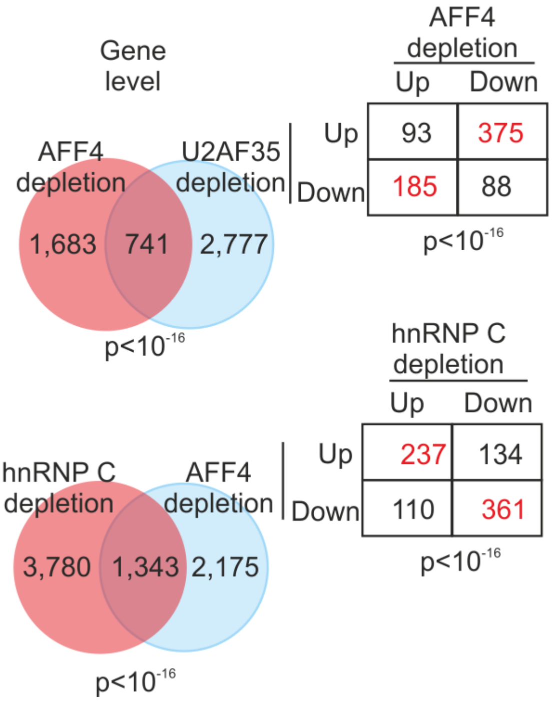

References for Supplementary Figures

1. Pacheco, T.R., Moita, L.F., Gomes, A.Q., Hacohen, N. and Carmo-Fonseca, M. (2006) RNA interference knockdown of hU2AF35 impairs cell cycle progression and modulates alternative splicing of Cdc25 transcripts. *Mol. Biol. Cell*, **17**, 4187-4199.
2. Pacheco, T.R., Coelho, M.B., Desterro, J.M., Mollet, I. and Carmo-Fonseca, M. (2006) In vivo requirement of the small subunit of U2AF for recognition of a weak 3' splice site. *Mol. Cell. Biol.*, **26**, 8183-8190.
3. Kralovicova, J. and Vorechovsky, I. (2010) Allele-dependent recognition of the 3' splice site of *INS* intron 1. *Hum. Genet.*, **128**, 383-400.
4. Lianoglou, S., Garg, V., Yang, J.L., Leslie, C.S. and Mayr, C. (2013) Ubiquitously transcribed genes use alternative polyadenylation to achieve tissue-specific expression. *Genes Dev.*, **27**, 2380-2396.
5. Zarnack, K., Konig, J., Tajnik, M., Martincorena, I., Eustermann, S., Stevant, I., Reyes, A., Anders, S., Luscombe, N.M. and Ule, J. (2013) Direct competition between hnRNP C and U2AF65 protects the transcriptome from the exonization of *Alu* elements. *Cell*, **152**, 453-466.
6. Luo, Z., Lin, C., Guest, E., Garrett, A.S., Mohaghegh, N., Swanson, S., Marshall, S., Florens, L., Washburn, M.P. and Shilatifard, A. (2012) The super elongation complex family of RNA polymerase II elongation factors: gene target specificity and transcriptional output. *Mol. Cell. Biol.*, **32**, 2608-2617.
